# Structure and fragmentation chemistry of the peptide radical cations of glycylphenylalanylglycine (GFG)

**DOI:** 10.1371/journal.pone.0308164

**Published:** 2024-08-13

**Authors:** Yinan Li, Justin Kai-Chi Lau, Teun van Wieringen, Jonathan Martens, Giel Berden, Jos Oomens, Alan C. Hopkinson, K. W. Michael Siu, Ivan K. Chu

**Affiliations:** 1 Department of Chemistry, University of Hong Kong, Hong Kong, China; 2 Department of Chemistry, York University, Toronto, ON, Canada; 3 Department of Chemistry and Biochemistry, University of Windsor, Windsor, ON, Canada; 4 FELIX Laboratory, Institute for Molecules and Materials, Radboud University, Nijmegen, The Netherlands; 5 Center for Mass Spectrometry Research and Clinical Application, Shandong Public Health Clinical Center Affiliated to Shandong University, Jinan, Shandong, China; Guru Ghasidas University Department of Pure & Applied Physics, INDIA

## Abstract

Herein, we explore the generation and characterization of the radical cations of glycylphenylalanylglycine, or [GFG]^•+^, formed via dissociative electron-transfer reaction from the tripeptide to copper(II) within a ternary complex. A comprehensive investigation employing isotopic labeling, infrared multiple-photon dissociation (IRMPD) spectroscopy, and density functional theory (DFT) calculations elucidated the details and energetics in formation of the peptide radical cations as well as their dissociation products. Unlike conventional aromatic-containing peptide radical cations that primarily form canonical π-radicals, our findings reveal that 75% of the population of the experimentally produced [GFG]^•+^ precursors are [GF_α_^•^G]^+^, where the radical resides on the middle α-carbon of the phenylalanyl residue. This unexpected isomeric ion has an enthalpy of 6.8 kcal/mol above the global minimum, which has an N-terminal captodative structure, [G_α_^•^FG]^+^, comprising 25% of the population. The [b₂-H]^•+^ product ions are also present in a ratio of 75/25 from [GF_α_^•^G]^+^/ [G_α_^•^FG]^+^, the results of which are obtained from matches between the IRMPD action spectrum and predicted IR absorption spectra of the [b₂-H]^•+^ candidate structures, as well as from IRMPD isomer population analyses.

## Introduction

Since the initial discovery that peptide radical cations can be made in the gas phase by collision-induced dissociation (CID) of the ternary [Cu^II^(L)(peptide)]^•2+^ complex [1], many of these cations have been observed and characterized [1–7]. The earliest studies [1–3] focused on peptides containing amino-acid residues with an aromatic side chain: tryptophan and tyrosine. The ease with which they were generated was attributed to the relatively low ionization energies of the corresponding amino acids (I.E.(Trp) = 7.3 eV; (Tyr) = 7.8 eV). In the presence of copper(II), tryptophan- and tyrosine-containing peptides can undergo oxidative electron-transfer dissociation. The resulting peptide radical cations are stabilized by effective dispersion of the charge and the spin, facilitated by the aromatic ring [6]. A number of studies have established that the initially generated peptide radical cations are π-radicals that can subsequently isomerize to other structures upon collisional activation [3–6]. Tryptophan-containing radical cations rearrange readily to give β-radicals that subsequently undergo N–C_α_ bond cleavage (see Scheme 1).

### Scheme 1

Attempts to create a radical cation from peptides containing a phenylalanine residue were less successful, perhaps due to relatively poor copper(II) binding to the phenyl ring of phenylalanine and its higher ionization energy (I.E.(Phe) = 8.7 eV). These features hinder the dissociative one-electron transfer reaction to Cu(II) that gives the initial π-radical cation structure typically observed in tryptophan-containing peptides. For example, CID of [Cu^II^(L)(GFG)]^•2+^ produces predominantly close-shell products, [b_2_]^+^ and [a_2_]^+^ ions, and only low abundance (≤5%) of the open-shell ion [GFG]^•+^ (c.f. References [1, 3]). It is this difference in the CID chemistry of the ternary complexes containing the phenylalanine, tryptophan and tyrosine residues that prompted us to conduct the current study on the structure of [GFG]^•+^. Peptide radical cations that contain only aliphatic residues are typically distonic with the radical residing on the N-terminal α-carbon rather than an α-carbon down the peptide chain [6, 8]. The preference for the N-terminal α-carbon is due to the especially favorable captodative effect offered by such placement of the radical [6]. Interconversion of the isomeric radical cations is prevented by relatively high barriers against isomerization [e.g., Reference [8]]. Collisional activation of these ions leads to cleavage of the peptide bond giving [b_n_−H]^•+^ ion.

Herein, we examine the structures of the [GFG]^•+^ ion, as the prototypical phenylalanine-containing peptide radical cation, using CID with extensive isotopic labelling with D (^2^H), ^18^O and ^15^N, and infrared multiple-photon dissociation (IRMPD) spectroscopy aided by density functional theory (DFT) calculations.

## Experimental section

### Materials and reagents

Fmoc-protected amino acids and chemicals were commercially available from Aldrich and Sigma (St. Louis, MO, USA), Bachem (King of Prussia, PA, USA), and GL Biochem (Shanghai, China). All of the studied peptides were prepared using solid-phase peptide synthesis in accordance with procedures described in the literature [9]. Cu^II^(Br_2_-terpy)(NO_3_)_2_ was synthesized by mixing Cu(NO_3_)_2_ with 6,6”-dibromo-2,2’:6’,2”-terpyridine (Br_2_-terpy) (1:1 ratio) in a boiling ethyl acetate solution. Cu^II^(12-crown-4)(ClO_4_)_2_ was prepared by mixing copper(II) perchlorate hexahydrate with 1,4,7,10-tetraoxacyclododecane (12-crown-4) (1:1 ratio) in a 1:1 water/methanol solution. Both Cu^II^(Br_2_-terpy)(NO_3_)_2_ and Cu^II^(12-crown-4)(ClO_4_)_2_ solutions were diluted with 1:1 water/methanol to a concentration of 600 μM before use.

### Mass spectrometry

A linear ion trap mass spectrometer (Thermo Fisher Scientific Inc., LTQ XL, Waltham, Massachusetts, USA) with an electrospray ionization (ESI) source was used. The ESI source was operated with nitrogen serving as both the curtain and collision gas. Sample solutions were infused at a flow rate of 180 μL/h by means of a syringe pump (Cole Parmer, Vernon Hills, IL, USA). A typical sample solution was prepared by mixing 50 μM peptide stock solution with 600 μM Cu^II^(Br_2_-terpy)(NO_3_)_2_ or 600 μM Cu^II^(12-crown-4)(ClO_4_)_2_ in a 1:1 ratio. Formation of [GFG]^•+^ occurred via dissociative electron transfer from the peptide to Cu^II^ after collisional activation of the [Cu^II^(L)(GFG)]^•2+^ ternary complex ion.

### Infrared Multiple-Photon Dissociation (IRMPD) spectroscopy

IRMPD spectroscopic experiments were performed at the Free-Electron Lasers for Infrared eXperiments (FELIX) laboratory at Radboud University, Nijmegen, the Netherlands [10] using a modified quadrupole ion-trap mass spectrometer (Bruker amaZon Speed ETD) providing optical access to the trapped ion population [11]. The ion of interest, e.g., [GFG]^•+^ or the [b_2_ –H]^•+^ ion, is mass-isolated and irradiated with a single pulse from FELIX, after which a mass spectrum is recorded. An IRMPD spectrum is obtained by plotting the photo-fragmentation yield defined as -ln[Σ*I*P/(Σ*I*P + Σ*I*F)], where *I*P and *I*F are the abundances of the precursor and fragment ions, respectively, as a function of IR frequency [12]. The laser pulse energy was carefully adjusted so as to avoid excessive precursor ion depletion. The IRMPD spectra were linearly corrected for the frequency-dependent laser pulse energy [13]. Structural identity of the trapped ion was obtained by matching the action spectrum with predicted IR absorption spectra of candidate structures using the B3LYP level of theory (see later).

Isomer population analyses were performed by parking the IR laser at selected frequencies while recording the precursor ion intensity as a function of the number of laser pulses as previously described [13]. When the precursor intensity did not converge to zero for a large number of pulses, multiple isomeric species were indicated in the ion population. By selecting IR frequencies that were absorbed by single isomers only, the relative population of the isomeric ions was thus determined.

### Computational methods

DFT calculations were conducted using the Gaussian 16 suite of programs [14]. Geometry optimizations were performed at the M06-2X/6-311++G(d,p) as well as the B3LYP/6-311++G(d,p) levels of theory [15, 16] To determine whether the optimized structures represented local minima or transition states on the potential energy surface (PES), harmonic frequency analyses were carried out, resulting in either no or one imaginary frequency. Intrinsic reaction coordinate (IRC) analyses [17] were employed to identify the two minima associated with each transition structure. All relative energies are expressed as relative enthalpy at 0 K (ΔH_0_°) and relative free energy at 298 K (ΔG_298_°) in parenthesis. The latter is included for readers who are interested in thermal equilibrium conditions. Predicted IR absorption spectra of candidate structures were calculated at the B3LYP/6-311++G(d,p) level [18–20] using the lowest-energy structures and after an anharmonicity correction with a scaling factor of 0.960.

## Results and discussion

### Fragmentation chemistry of [GFG]^•+^

**Fig 1:**
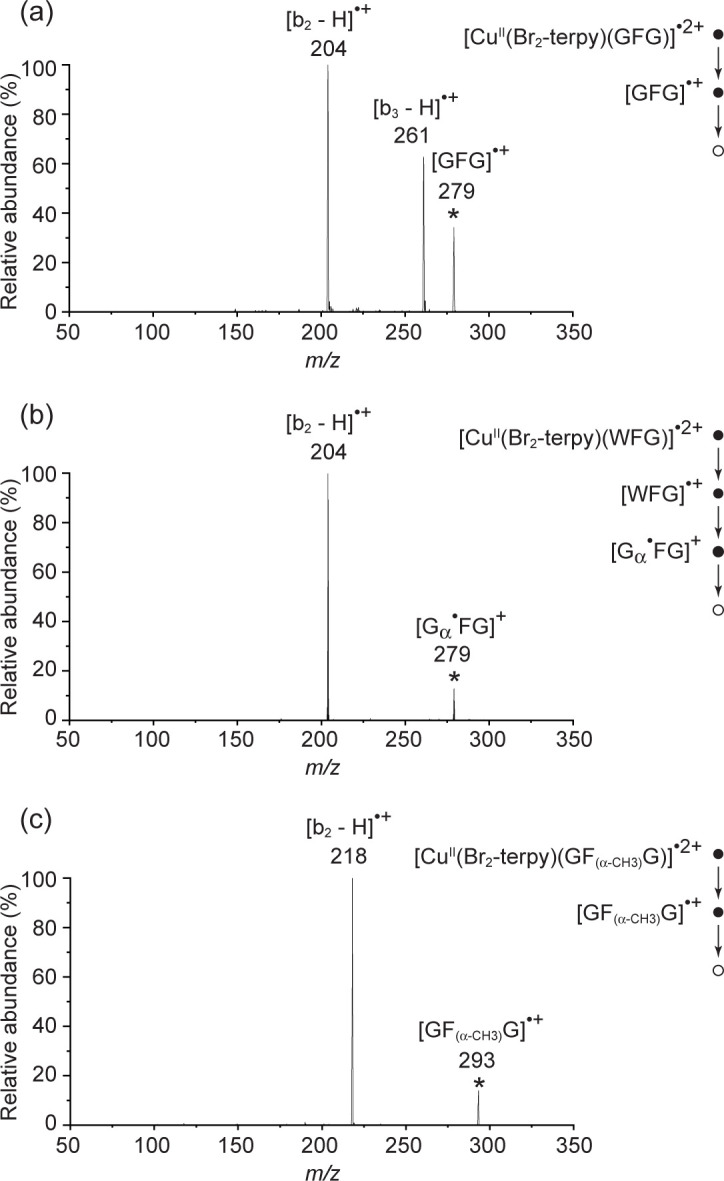
(a) CID of [GFG]^•+^ from [Cu^II^(Br_2_-terpy)(GFG)]^•2+^, * = precursor ion; (b) CID of [G_α_^•^FG]^+^ formed from [Cu^II^(Br_2_-terpy)(WFG)]^•2+^; and (c) CID of [Cu^II^(Br_2_-terpy)(GF_(α-CH3)_G)]^•2+^.

The CID of [Cu^II^(Br_2_-terpy)(GFG)]^•2+^ via dissociative electron transfer gives [GFG]^•+^ that, in turn, readily fragments to yield b-type product ions (Fig 1A). Formation of these b-type product ions are largely driven by proton mobility, as exemplified by the prototypical tripeptide triglycine [8] (see also S1 Fig). Fig 1A shows the [b_2_ –H]^•+^ product ion as the base peak with a second product of [b_3_ –H]^•+^. It is of note parenthetically that the relative abundance between [b_3_ –H]^•+^ and [b_2_ –H]^•+^ is dependent on the nature of the auxiliary ligand (L) with Br_2_-terpy giving the most at ~60%, me_5_dien (N,N,N^I^,N^II^,N^II^-pentamethyldiethylenetriamine) at ~45%, dien at ~25%, terpy at ~20%, 4Cl-terpy at ~20%, and 12-crown-4 at ~3%.

Fragmentation of the isomeric tryglycine radical cations (Table 1) is instructive [8]:

**Table 1:** Fragmentation of the triglycine radical cations with the radical located at different α-carbons of the peptide.

Precursor ion	Product ions observed
[G_α_^•^GG]^+^	[b_2_ –H]^•+^
[GG_α_^•^G]^+^	[b_2_ –H]^•+^, [b_3_ –H]^•+^
[GGG_α_^•^]^+^	[b_2_]^+^, [b_2_ –H]^•+^, [b_3_ –H]^•+^

**Fig 2:**
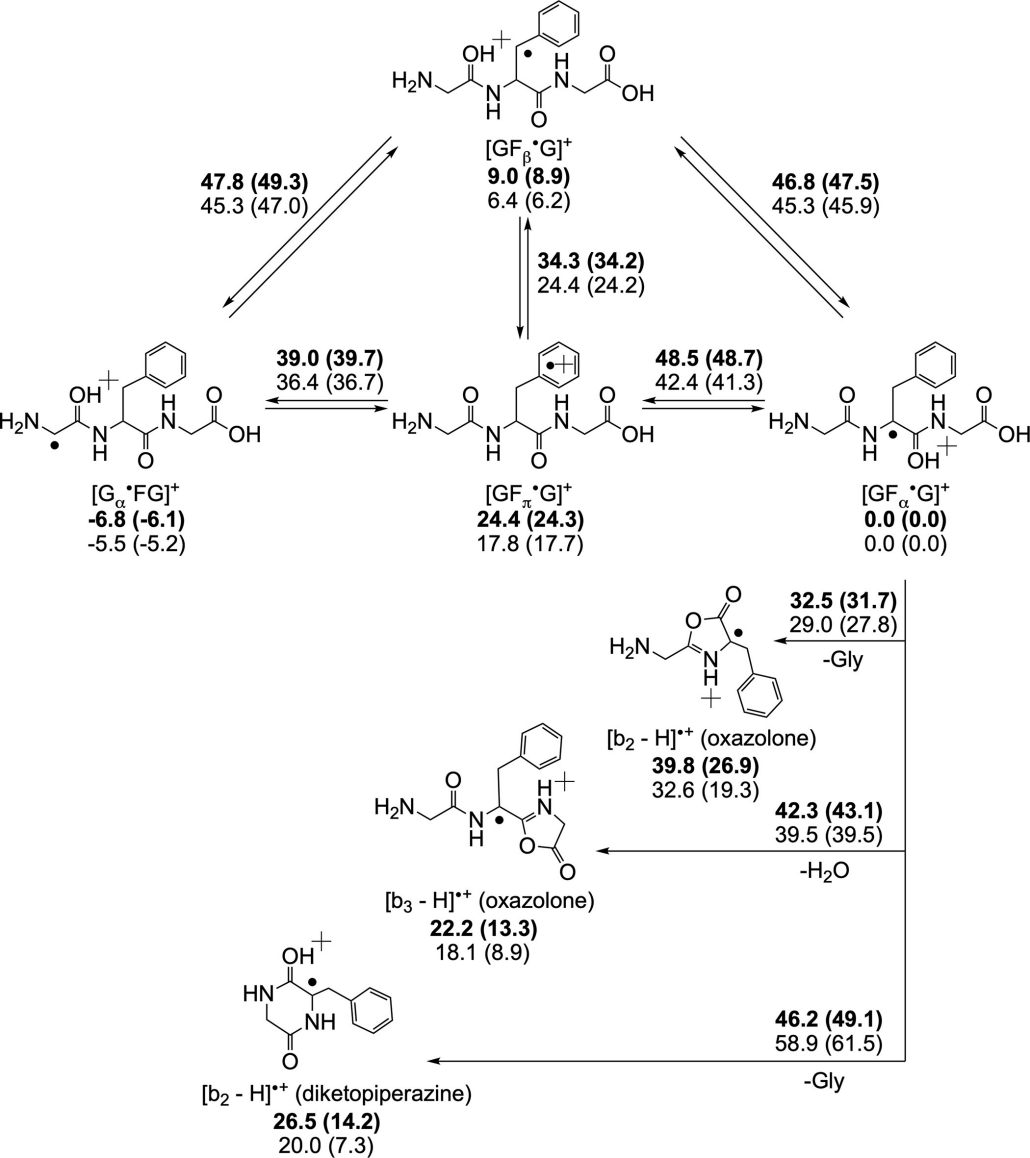
Isomers of [GFG]^•+^ and dissociation products: Upper numbers and in bold, M06-2X/6-311++G(d,p); lower numbers and in regular font, B3LYP/6-311++G(d,p). All relative energies are in kcal mol^-1^ and shown in the following format: ΔH_0_° (ΔG_298_°).

N.B. Location of the radical symbol ^**•**^ within the square brackets indicates the radical site; when it is outside of the square bracket, its location is undefined. The subscript α denotes the location of the radical on the specific α-carbon, as detailed in the proposed nomenclature system for peptide ion fragmentation [21]. The [b_3_ –H]^•+^ ion is not a product of [G_α_^•^GG]^+^, the global minimum structure with the radical residing on the N-terminal alpha carbon (C_α_), but from non-convertible higher-energy tripeptide isomers that have the radical residing on downstream C_α_. Fig 1B shows that this is also true for the tripeptide GFG, where [G_α_^•^FG]^+^ was formed via the CID of [Cu^II^(Br_2_-terpy)WFG]^•2+^ in a process analogous to that of triglycine [8]. ^18^O- and ^15^N-labelling experiments (S2 Fig) show that the oxygen loss (as water) from [GFG]^•+^ to give the [b_3_ –H]^•+^ ion did not originate from the first or the second peptide linkage, and that collisional activation of [b_3_ –H]^•+^ resulted in the loss of ammonia from the N-terminus, not water, which implies that [b_3_ –H]^•+^ does not have an oxazolone structure, irrespective of this placeholder assignment as a possible product in Fig 2. Chemical intuition leads us to suggest that [b_3_ –H]^•+^ is plausibly formed via nucleophilic attack by the N-terminal nitrogen on the C-terminal protonated carboxylic carbon, thereby giving a macrocyclic radical cation [22] that subsequently rearranges to give a diketopiperazine structure. Proton transfer followed by cyclization results in elimination of the terminal nitrogen as ammonia.

**Fig 3:**
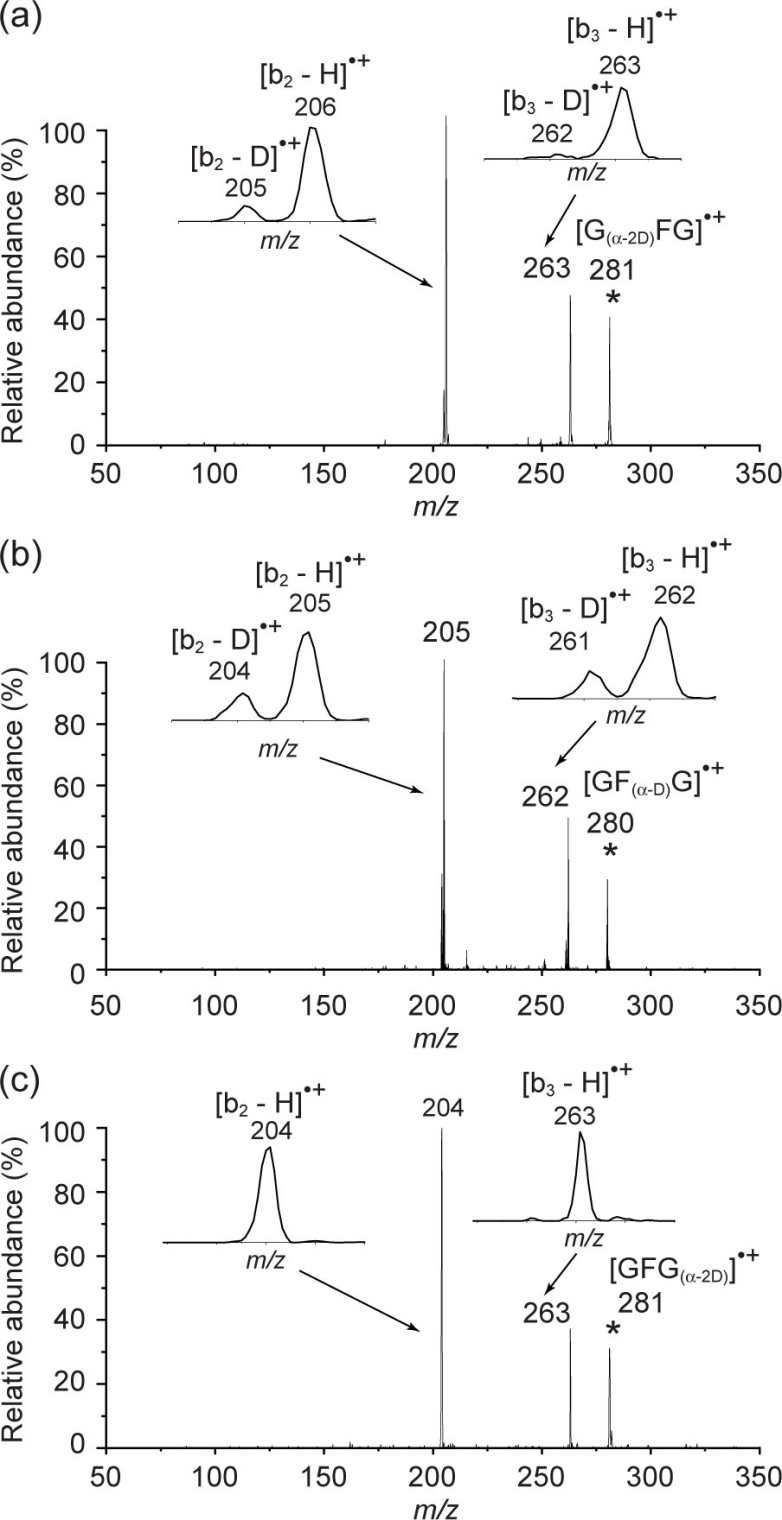
CID of the D-labelled peptides: (a) [G_(α-2D)_FG]^•+^, * = precursor ion; (b) [GF_(α-D)_G]^•+^; and (c) [GFG_(α-2D)_]^•+^.

D-labelling experiments of all three C_α_ (Fig 3) show some extent of H/D scrambling, which is not as informative as we were hoping it would be to determine the source of the hydrogen loss and hence the plausible [GFG]^•+^ structure. CID of [GF_(α-CH3)_G]^•+^, in which the C_α_ of the phenylalanine residue is methylated and hence cannot be the radical site, results in only the observation of [b_2_ –H]^•+^ and none of [b_3_ –H]^•+^ (Fig 1C). Placement of the radical on the C_α_ of the first glycyl residue can result in [b_2_ –H]^•+^ and none of [b_3_ –H]^•+^, using the CID of [GGG]^•+^ as the guide (see above) and also the CID of [G_α_^•^FG]^+^ as the experimental proof (Fig 1B). Placing the radical on the C_α_ of the third and last glycyl residue is expected to lead to the b_2_^+^, [b_2_ –H]^•+^, and [b_3_ –H]^•+^ ion (see above for triglycine). So the evidence points to the C_α_ of the first residue being the radical site, when the second residue is unable to accommodate the radical.

DFT calculations on the [GFG]^•+^ system, the results of which are summarized in Fig 2, show that the isomer with the radical on the first C_α_, or [G_α_^•^FG]^+^, is the global minimum structure at both the M06-2X and the B3LYP levels of theory. As the former, by consensus, gives energies that are more comparable with experiments (especially with respect to reaction barriers) and that both M06-2X and B3LYP give the same trends [23–26], we will restrict our discussion on energetics using values calculated only by means of M06-2X. The isomer with the radical situated on the C_α_ of the phenylalanyl residue, [GF_α_^•^G]^+^, is higher in enthalpy at 0 K by 6.8 kcal/mol. This isomer, once formed, is stable against isomerization; the barrier against dissociation to [b_2_ –H]^•+^ is only 32.5 kcal/mol, lower by > 10 kcal/mol versus the isomerization barriers to other possible [GF^•^G]^+^ isomers.

As alluded to earlier, CID of the ternary [Cu^II^(L)(GWG)]^•2+^ complex gives a peptide ion that is a π-radical, but not that of [Cu^II^(L)(GFG)]^•2+^. This difference is attributable to the higher ionization energy of F versus W. We have examined in detail how [GGW]^•+^ is produced from [Cu^II^(dien)(GGW)]^•2+^ after collisional activation [27]. The ternary complex is initially coordinated through the carboxylate anion (-COO^-^) and the dissociation requires the proton to migrate from the terminal -NH_3_^+^ to the -COO^-^. This leads to a high-energy intermediate (IM1 in Reference [27]) in which the copper is coordinated to the π-system of the tryptophan residue; subsequent homolytic fission gives the peptide π-radical cation. The phenyl group in phenylalanine is a much weaker π-donor versus the indole group (in tryptophan), chemical intuition suggests that the analogous high-energy intermediate is formed via a different basic group, either an amide oxygen or the N-terminal nitrogen. Dissociation and proton transfer then give the α-radical peptide cation.

### IRMPD spectroscopy

**Fig 4:**
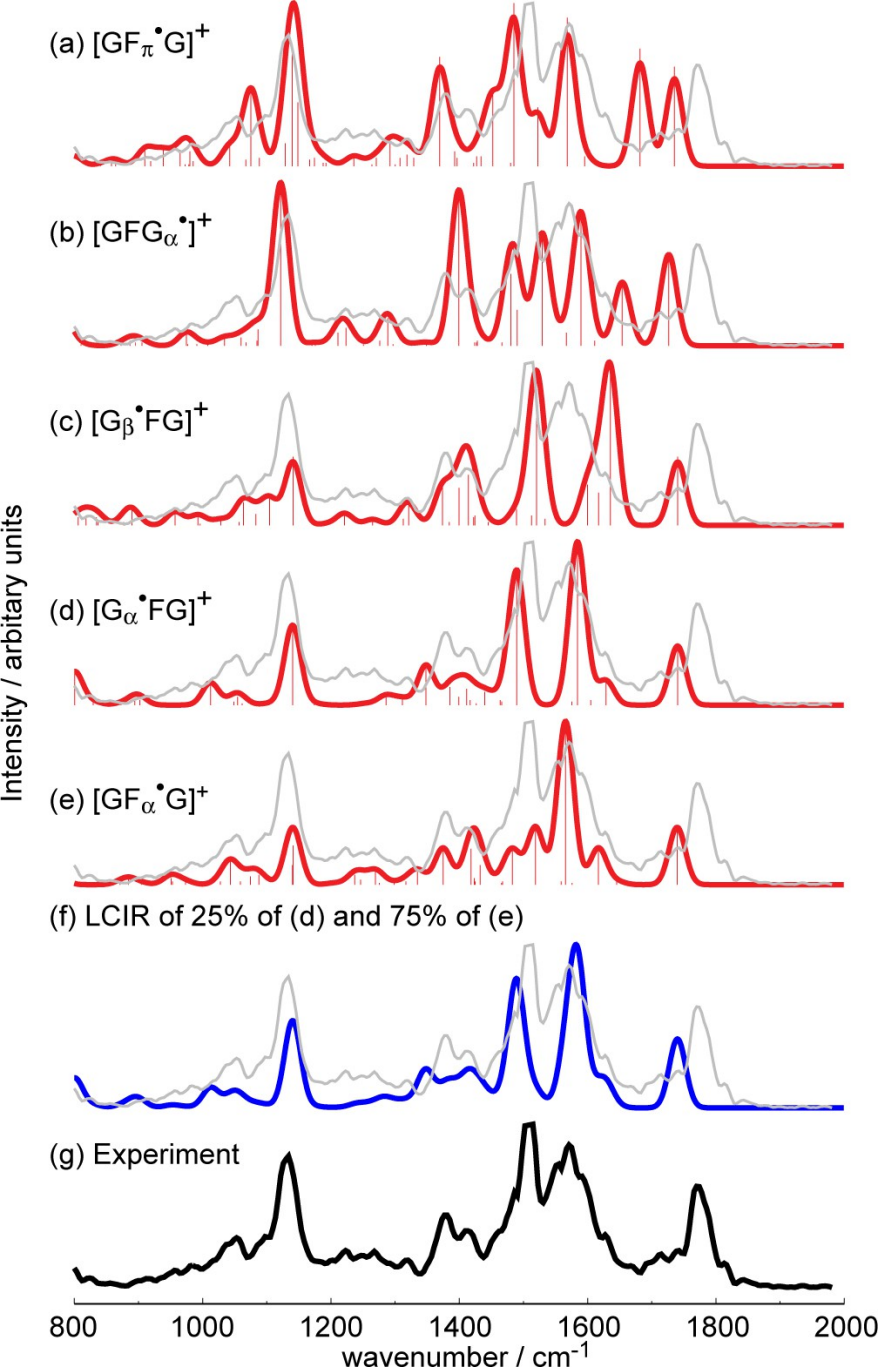
Predicted IR absorption spectra of candidate structures of [GFG]^•+^ and the IRMPD action spectrum of [GFG]^•+^: (a) [GF_π_^•^G]^+^; (b) [GFG_α_^•^]^+^; (c) [G_β_^•^FG]^+^; (d) [G_α_^•^FG]^+^; (e) [GF_α_^•^G]^+^; (f) linear combination of the IR absorption spectra (LCIR) of 25/75 [G_α_^•^FG]^+^/ [GF_α_^•^G]^+^; and (g) IRMPD action spectrum.

The IRMPD action spectra coupled with a comparison with the predicted IR absorption spectra of candidate structures from the [GFG]^•+^ family are valuable in revealing the [GFG]^•+^ structures formed in the dissociation of [Cu^II^(Br_2_-terpy)(GFG)]^•2+^ (Fig 4). The IR absorption spectra of the candidate structures shown in Fig 4 were all determined using B3LYP, which by consensus gives spectra that are comparable to experiments [28–32]. It is readily apparent that none of the IR absorption spectra of individual structures fit all the features in the IRMPD action spectrum (the experiment). However, a linear combination of the IR absorption spectra (Fig 4F) comprising 25% of [G_α_^•^FG]^+^ (Fig 4D) and 75% of [GF_α_^•^G]^+^ (Fig 4E), the two lowest-energy isomers of the [GFG]^•+^ family, fits the IRMPD action spectrum (Fig 4G) reasonably well. Our hypothesis is that the [GF_α_^•^G]^+^ isomer, once formed after CID of [Cu^II^(Br_2_-terpy)(GFG)]^•2+^, is stable and does not convert to the lowest-energy isomer—[G_α_^•^FG]^+^—owing to the fact that the isomerization barriers are higher than the barriers against dissociations (Fig 2).

**Fig 5:**
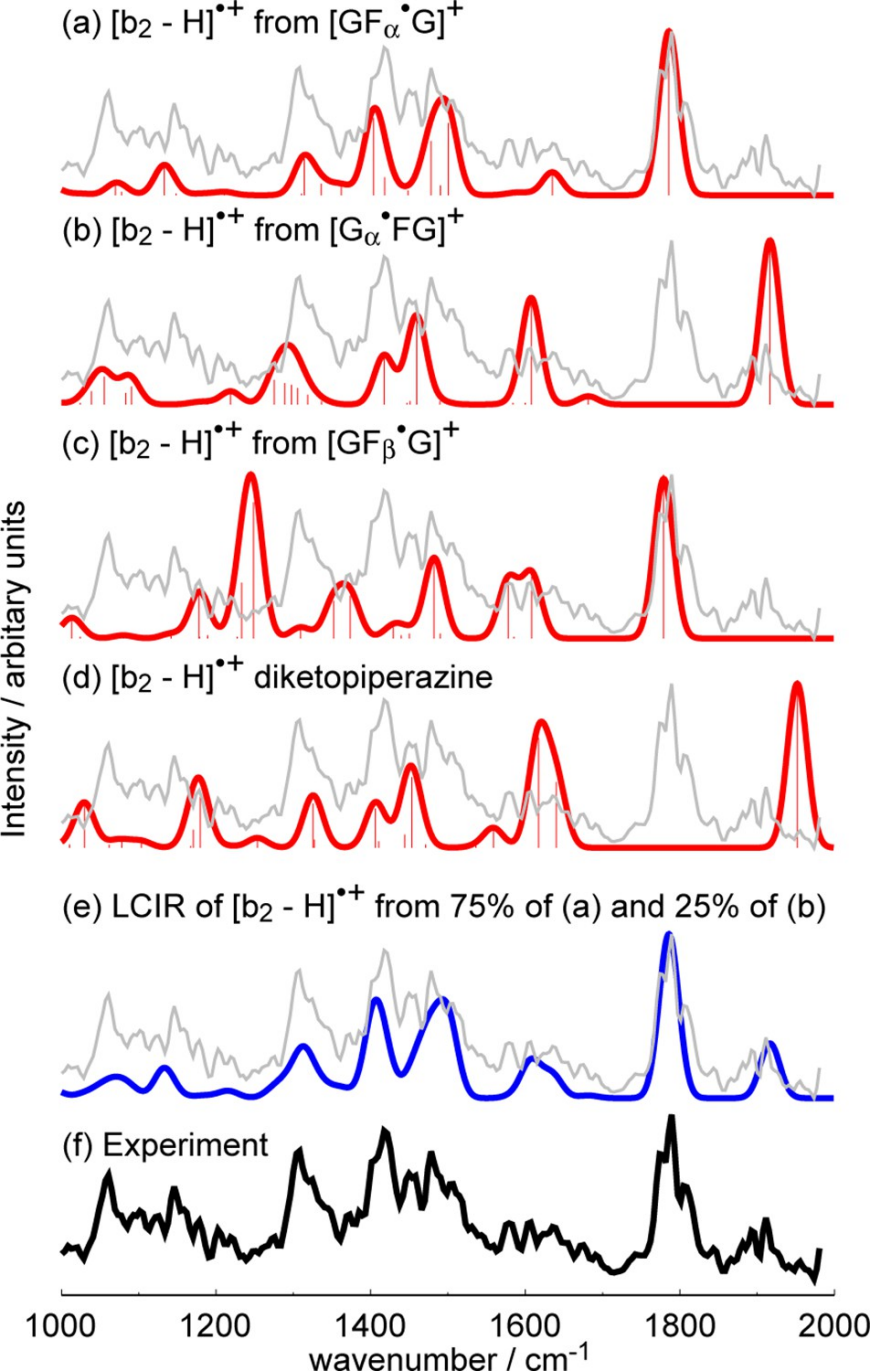
Predicted IR absorption spectra of candidate structures of [b_2_ –H]^•+^ and the IRMPD action spectrum of [b_2_ –H]^•+^: (a) from [GF_α_^•^G]^+^; (b) from [G_α_^•^FG]^+^; (c) from [GF_β_^•^G]^+^; (d) diketopiperazine; (e) LCIR of 75/25 the [b_2_ –H]^•+^ from [GF_α_^•^G]^+^/ [G_α_^•^FG]^+^; and (f) IRMPD action spectrum.

**Fig 6:**
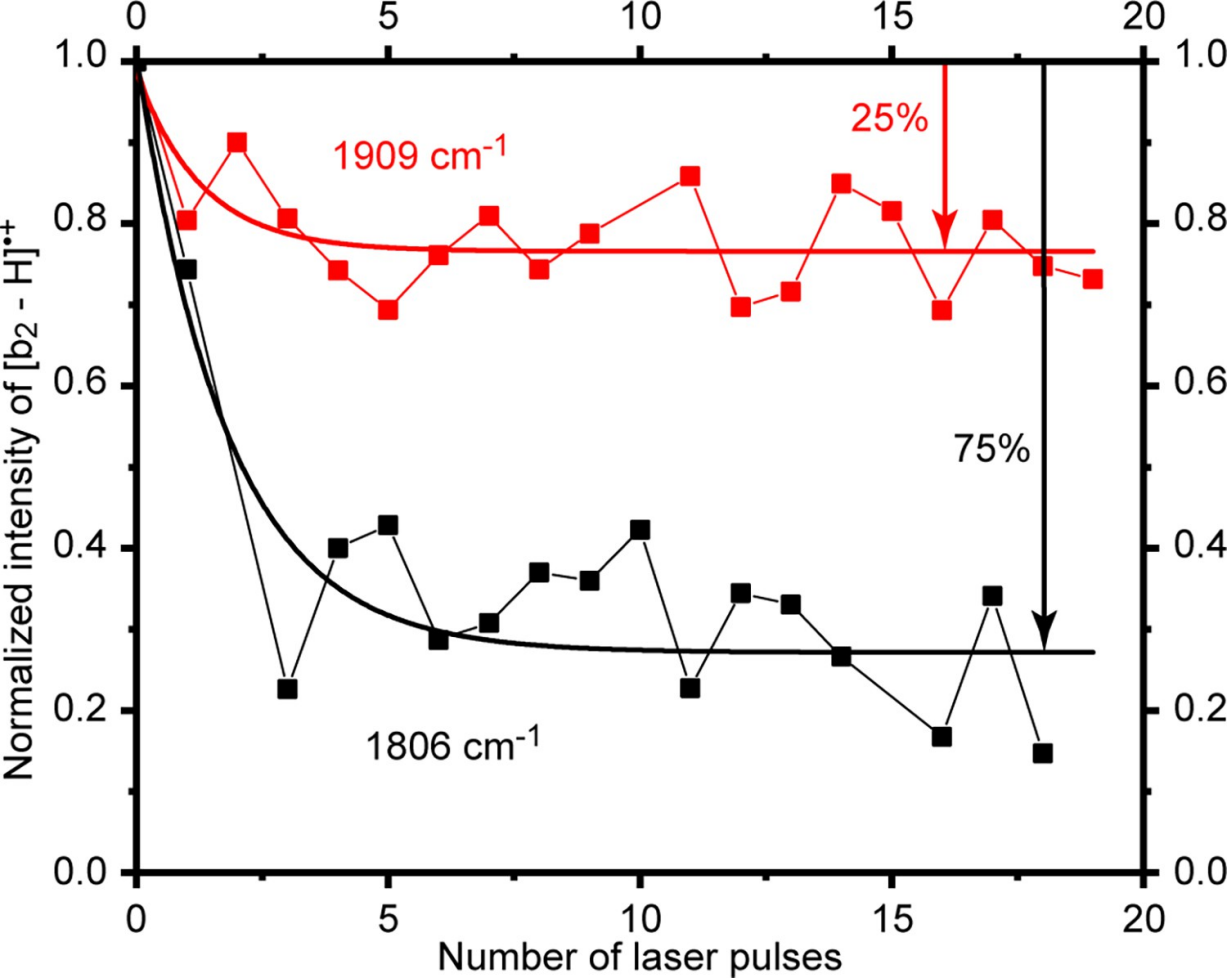
[b_2_ –H]^•+^ isomer population analysis: Black trace, parking the IR laser at 1806 cm^-1^ and red trace, parking at 1909 cm^-1^; the plateau regions show a decrease in [b_2_ –H]^•+^ ion intensity by 75% and 25%, respectively, meaning that the [b_2_ –H]^•+^ isomer that absorbs at 1806 cm^-1^ (from [GF_α_^•^G]^+^) is present at 75% of the population, and that the isomer that absorbs at 1909 cm^-1^ (from [G_α_^•^FG]^+^) is present at 25%.

Fig 5 shows a comparison of the IRMPD action spectrum of [b_2_ –H]^•+^, the major dissociation product of [GFG]^•+^, with the predicted IR absorption spectra of candidate [b_2_ –H]^•+^ structures. The best-fitting candidate structures are all oxazolones, and the observed [b_2_ –H]^•+^ is also a mixture comprising 75% of the [b_2_ –H]^•+^ from [GF_α_^•^G]^+^ (Fig 5A) and 25% of the [b_2_ –H]^•+^ from [G_α_^•^FG]^+^ (Fig 5B). A linear combination of the predicted IR absorption spectra of the two (Fig 5E) provided the best fit to the IRMPD action spectrum (Fig 5F). Isomer population analyses by parking the IR laser at 1806 cm^-1^ and subsequently at 1909 cm^-1^ resulted in precursor ion depletion curves (13) that plateaued at large number of laser pulses, thereby strongly suggesting the presence of a non-absorbing isomer and in the ratio of 3 to 1 between the two experiments (Fig 6). The [b_2_ –H]^•+^ from [GF_α_^•^G]^+^ has a strong predicted IR absorption band at ~ 1800 cm^-1^ (Fig 5A), while that from [G_α_^•^FG]^+^ one at ~ 1900 cm^-1^ (Fig 5B). Parking the IR laser at 1806 cm^-1^ would presumably dissociate the [b_2_ –H]^•+^ from [GF_α_^•^G]^+^ comprising 75% of the relative population; similarly, illuminating at 1909 cm^-1^ would dissociate the [b_2_ –H]^•+^ from [G _α_^•^FG]^+^ comprising 25% of the isomer population. These predictions are reflected by the results of isomer population analyses (Fig 6).

## Conclusion

Collision-induced dissociation of the ternary complex, [Cu^II^(L)(GFG)]^•2+^, unlike that of [Cu^II^(L)(GWG)]^•2+^ and [Cu^II^(L)(GYG)]^•2+^, results in GFG α-radical cations due to the high ionization energy of the phenylalanine sidechain and poor Cu binding to the phenyl ring. IRMPD spectroscopy shows that the GFG radical cations thus formed are a mixture of 75% [GF_α_^•^G]^+^ and 25% [G_α_^•^FG]^+^; the former isomer once formed is stable against isomerization, as their activation barriers are all significantly above the dissociation barriers to give [b_2_ –H]^•+^. IRMPD spectroscopy also shows that the [b_2_ –H]^•+^ ions are a 75/25 mixture of those from [GF_α_^•^G]^+^/ [G_α_^•^FG]^+^. Isomer population analyses provide independent support for the above conclusion.

## Supporting information

S1 Fig Product ion spectra of (a) [GGG]^•+^, (b) [GGA]^•+^; (c) [GLG]^•+^; (d) [GLA]^•+^; (e) [GWG]^•+^; (f) [GHG]^•+^; (g) [GMG]^•+^; and (h) [GFG]^•+^. All precursor ions were formed from dissociative electron transfer reactions of [Cu^II^(12-crown-4)(peptide)]^•2+^.
(PDF)

S2 Fig Product ion spectra of (a) [G_(18O)_FG]^•+^; (b)] GF_(18O)_G]^•+^; and (c) [G_(15N)_FG]^•+^. Inserts show the corresponding product ion spectra of [b_3_ –H]^•+^.
(PDF)

S1 File Cartesian coordinates of the structures shown in Fig 2. (PDF)

S1 Scheme (TIF)
