# Supplementary material for: Structure and fragmentation chemistry of the peptide radical cations of glycylphenylalanylglycine (GFG)
Source: PLoS One. 2024 Aug 13;19(8):e0308164. doi: 10.1371/journal.pone.0308164 (PMC11321575; doi:10.1371/journal.pone.0308164)

Figure S1. Product ion spectra of (a) $[GGG]^{\bullet+}$, (b) $[GGA]^{\bullet+}$; (c) $[GLG]^{\bullet+}$; (d) $[GLA]^{\bullet+}$; (e) $[GWG]^{\bullet+}$; (f) $[GHG]^{\bullet+}$; (g) $[GMG]^{\bullet+}$; and (h) $[GFG]^{\bullet+}$. All precursor ions were formed from dissociative electron transfer reactions of $[Cu^{II}(12\text{-crown-4})(\text{peptide})]^{\bullet 2+}$.

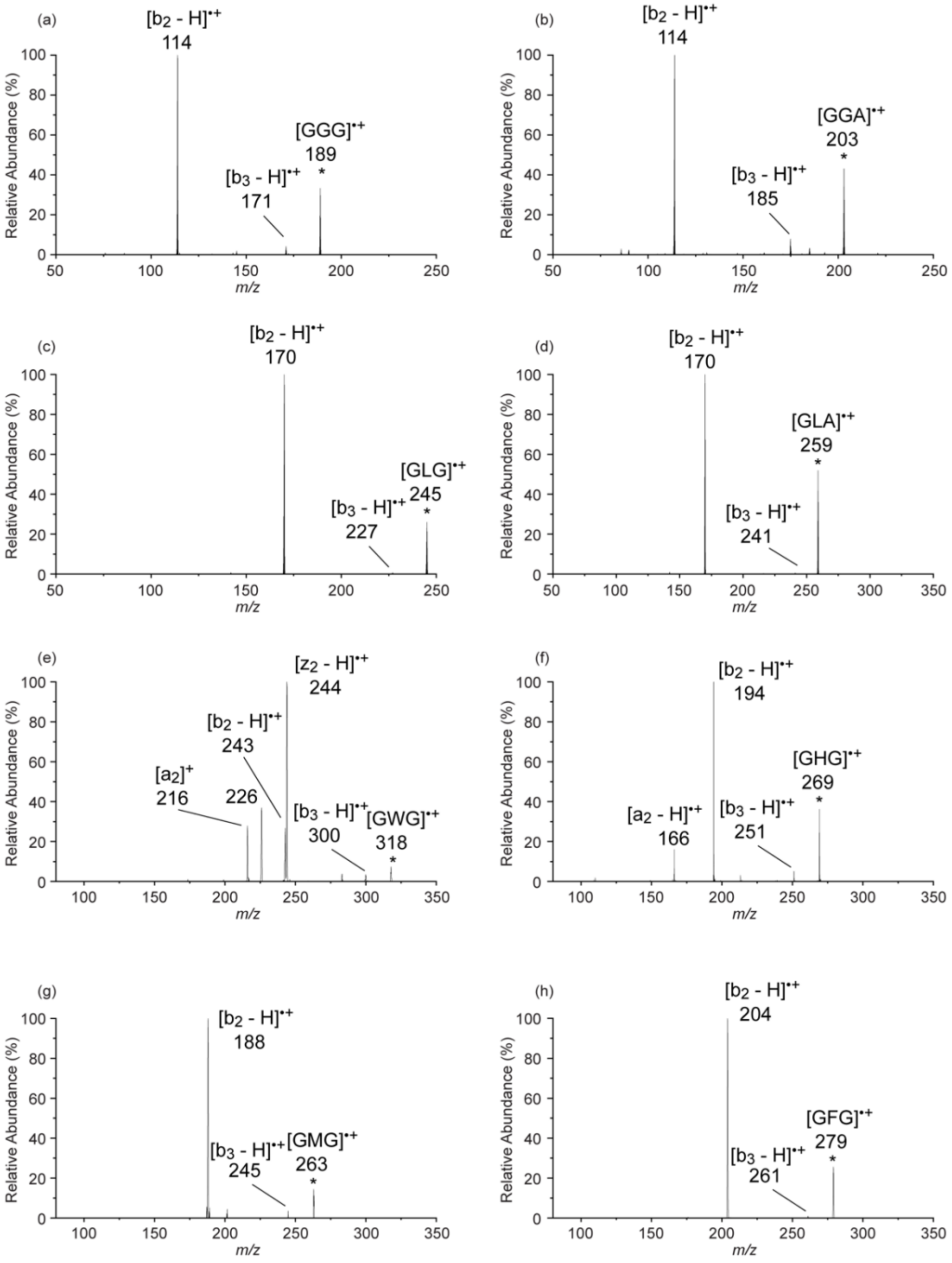

Supplement: S1 Fig — Product ion spectra of (a) [GGG]•+, (b) [GGA]•+; (c) [GLG]•+; (d) [GLA]•+; (e) [GWG]•+; (f) [GHG]•+; (g) [GMG]•+; and (h) [GFG]•+. All precursor ions were formed from dissociative electron transfer reactions of [CuII(12-crown-4)(peptide)]•2+. (PDF) [file pone.0308164.s001.pdf]
