# Supplementary material for: Structure and fragmentation chemistry of the peptide radical cations of glycylphenylalanylglycine (GFG)
Source: PLoS One. 2024 Aug 13;19(8):e0308164. doi: 10.1371/journal.pone.0308164 (PMC11321575; doi:10.1371/journal.pone.0308164)

Figure S2. Product ion spectra of (a) $[G_{(18O)}FG]^{\bullet+}$; (b) $]GF_{(18O)}G]^{\bullet+}$; and (c) $[G_{(15N)}FG]^{\bullet+}$. Inserts show the corresponding product ion spectra of $[b_3 – H]^{\bullet+}$.

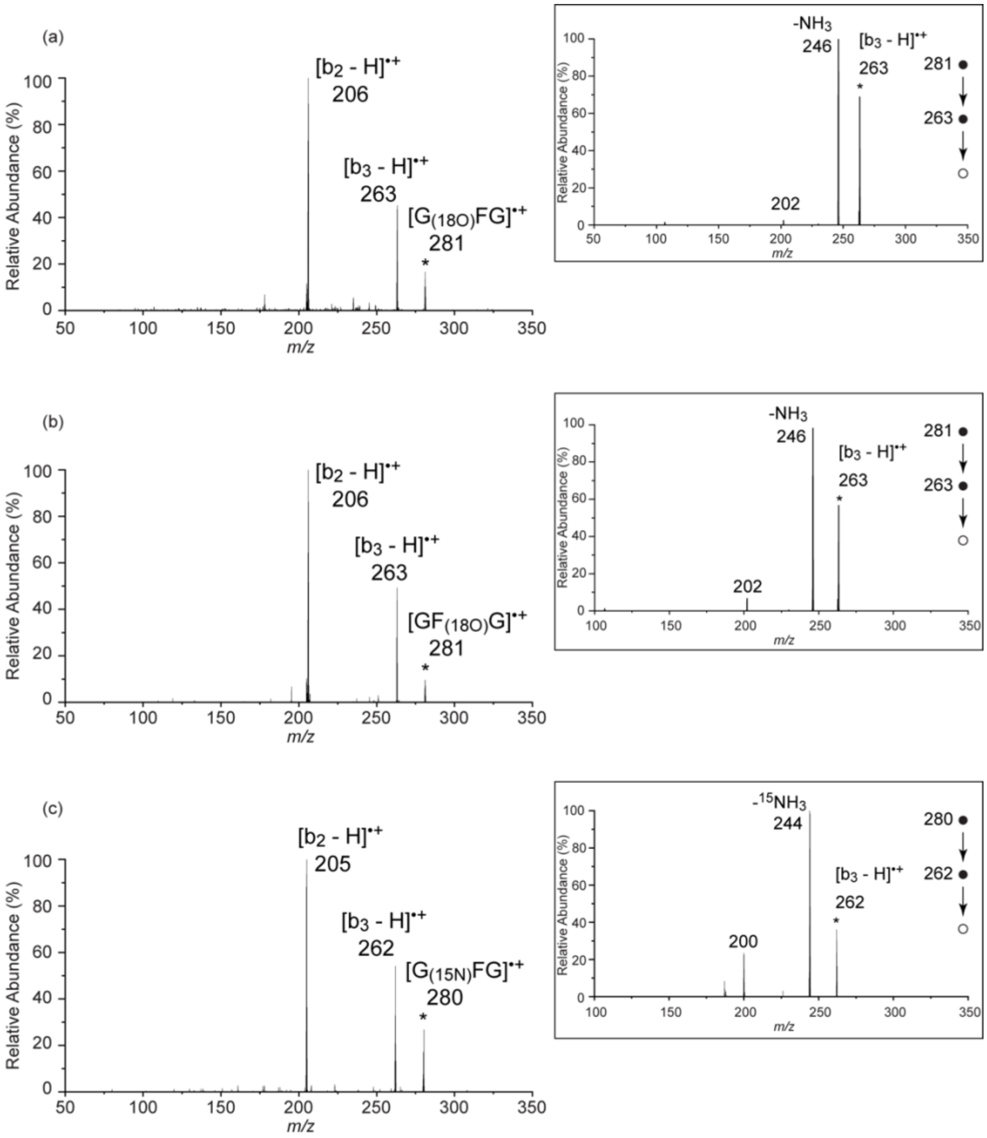

Supplement: S2 Fig — Product ion spectra of (a) [G(18O)FG]•+; (b)] GF(18O)G]•+; and (c) [G(15N)FG]•+. Inserts show the corresponding product ion spectra of [b3 –H]•+. (PDF) [file pone.0308164.s002.pdf]
