# Supplementary material for: Structure and fragmentation chemistry of the peptide radical cations of glycylphenylalanylglycine (GFG)
Source: PLoS One. 2024 Aug 13;19(8):e0308164. doi: 10.1371/journal.pone.0308164 (PMC11321575; doi:10.1371/journal.pone.0308164)
Supplement: S1 File — (PDF) [file pone.0308164.s003.pdf]

Cartesian coordinates of the structures shown in Figure 2.

B3LYP/6-311++G(d,p)

$[GF_{\alpha}{}^{\bullet}G]^{+}$

N	-3.512815	1.899445	-0.000001
C	-2.574946	3.014660	0.000011
H	-4.106416	1.884801	-0.819698
H	-4.106409	1.884777	0.819700
C	-1.121474	2.554284	0.000006
H	-2.688709	3.664089	-0.873209
H	-2.688708	3.664071	0.873244
O	-0.203383	3.376221	0.000016
N	-0.961050	1.191278	-0.000010
H	-1.885929	0.728952	-0.000015
C	0.135830	0.360420	-0.000021
C	-0.132354	-1.136946	-0.000051
C	1.500595	0.822729	-0.000012
H	0.361093	-1.573910	-0.877397
H	0.361139	-1.573954	0.877246
C	-1.588706	-1.553806	-0.000021
O	1.880169	2.065861	0.000013
C	-2.266703	-1.761734	-1.206565
C	-2.266653	-1.761733	1.206551
H	-1.747376	-1.626989	-2.150292
C	-3.600673	-2.164350	-1.206457
C	-3.600622	-2.164350	1.206499
H	-1.747286	-1.626989	2.150257
H	-4.111010	-2.336771	-2.146799
C	-4.269348	-2.365283	0.000035
H	-4.110920	-2.336770	2.146863
N	2.486738	-0.072755	-0.000030
H	2.305272	-1.071660	-0.000045
C	3.898305	0.281494	-0.000013
C	4.686884	-1.017913	0.000009
H	4.159043	0.875827	-0.880066
H	4.159017	0.875844	0.880035
O	4.164596	-2.105184	0.000015
O	5.997235	-0.786661	0.000021
H	6.472638	-1.633600	0.000036
H	1.077383	2.721458	0.000016
H	-5.302698	-2.691684	0.000057

$[G_{\alpha}{}^{\bullet}FG]^{+}$

N	2.953892	3.798342	-0.420276
C	2.477924	2.645699	-0.914118
H	3.792063	4.220797	-0.787751
H	2.472345	4.264022	0.337675
C	1.309013	2.070588	-0.376824
H	3.014205	2.175685	-1.726170
O	0.771471	2.654380	0.660550
N	0.752337	0.972316	-0.944707
H	1.249677	0.608880	-1.745815
C	-0.129796	-0.016985	-0.289497
H	-0.321988	-0.762374	-1.062338
C	0.542855	-0.727994	0.920158
C	-1.465272	0.606432	0.134464
H	0.722491	0.001551	1.713217
H	-0.180932	-1.449165	1.313296
C	1.831408	-1.425369	0.540910
O	-1.507776	1.689395	0.745097
C	3.065412	-0.920486	0.962234

M06-2X/6-311++G(d,p)

$[GF_{\alpha}{}^{\bullet}G]^{+}$

N	-3.507275	1.725447	-0.000023
C	-2.614716	2.871831	-0.000004
H	-4.098675	1.686462	-0.819680
H	-4.098689	1.686445	0.819623
C	-1.152507	2.452734	0.000002
H	-2.744988	3.514464	-0.874516
H	-2.745001	3.514444	0.874520
O	-0.255973	3.289698	0.000017
N	-0.964725	1.099444	-0.000010
H	-1.879482	0.618409	-0.000019
C	0.144657	0.295512	-0.000009
C	-0.096672	-1.201310	-0.000026
C	1.494413	0.788862	0.000000
H	0.392679	-1.634294	-0.880977
H	0.392706	-1.634318	0.880896
C	-1.558819	-1.579223	-0.000008
O	1.839485	2.033675	0.000014
C	-2.246813	-1.726289	-1.205005
C	-2.246784	-1.726283	1.205006
H	-1.715555	-1.624488	-2.146226
C	-3.607265	-2.016120	-1.204906
C	-3.607236	-2.016113	1.204940
H	-1.715505	-1.624477	2.146214
H	-4.130846	-2.143617	-2.144602
C	-4.288599	-2.158471	0.000026
H	-4.130795	-2.143605	2.144650
N	2.493077	-0.083679	-0.000005
H	2.335695	-1.086865	-0.000016
C	3.885940	0.322772	0.000005
C	4.716487	-0.944899	-0.000001
H	4.123445	0.922671	-0.882010
H	4.123436	0.922656	0.882033
O	4.233604	-2.043453	-0.000013
O	6.010899	-0.670013	0.000007
H	6.515918	-1.497148	0.000003
H	1.017019	2.673351	0.000018
H	-5.345534	-2.395844	0.000039

$[G_{\alpha}{}^{\bullet}FG]^{+}$

N	3.133429	3.358653	-0.354984
C	2.581250	2.275082	-0.906694
H	4.020007	3.718260	-0.672056
H	2.652630	3.842399	0.392989
C	1.344289	1.808324	-0.425371
H	3.103440	1.770054	-1.706864
O	0.832217	2.408388	0.605543
N	0.697855	0.786213	-1.032691
H	1.192961	0.375681	-1.813250
C	-0.202998	-0.146471	-0.338300
H	-0.413937	-0.932899	-1.064306
C	0.448688	-0.782467	0.914657
C	-1.506349	0.539804	0.056537
H	0.527667	-0.031681	1.704650
H	-0.223323	-1.566498	1.275224
C	1.810176	-1.340007	0.582433
O	-1.496238	1.646105	0.616542
C	2.963652	-0.683017	1.009741

C	1.810151	-2.587853	-0.239859
H	3.098952	-0.037907	1.593417
C	4.254008	-1.558572	0.606716
C	2.995703	-3.224386	-0.599254
H	0.862269	-3.017603	-0.551450
H	5.202153	-1.164712	0.954324
C	4.221516	-2.708456	-0.178565
H	2.962653	-4.129812	-1.193928
N	-2.564218	-0.096615	-0.126928
H	-2.532629	-0.972147	-0.640031
C	-3.893539	0.318801	0.296685
C	-4.877418	-0.740882	-0.165346
H	-3.946975	0.426136	1.383357
H	-4.161783	1.286238	-0.136233
O	-4.551065	-1.728588	-0.776168
O	-6.124382	-0.431927	0.190706
H	-6.724971	-1.128545	-0.120414
H	-0.202806	2.320684	0.813899
H	5.143413	-3.209333	-0.449394

$[GF_{\pi}{}^{\bullet}G]^{+}$

N	1.702245	4.093500	-0.137565
C	2.626763	3.048905	-0.481193
H	1.507997	4.760071	-0.871259
H	1.879784	4.560660	0.740532
C	1.950785	1.649718	-0.494168
H	3.058283	3.178695	-1.476431
H	3.460423	2.971975	0.221529
O	2.613615	0.648192	-0.799257
N	0.666112	1.613379	-0.158183
H	0.236698	2.488736	0.154462
C	-0.155144	0.422374	-0.154912
H	-0.176755	0.001074	-1.165100
C	0.357625	-0.668088	0.837724
C	-1.578658	0.888490	0.245802
H	0.424023	-0.227399	1.834595
H	-0.407394	-1.450907	0.881655
C	1.663183	-1.306324	0.454656
O	-1.746316	1.972461	0.780994
C	2.761198	-1.290284	1.354845
C	1.771242	-2.081204	-0.737816
H	2.680858	-0.722057	2.274493
C	3.909744	-1.996307	1.071857
C	2.923612	-2.783397	-1.018505
H	0.934369	-2.122393	-1.425645
H	4.743023	-1.985456	1.763744
C	3.999908	-2.741440	-0.119487
H	2.999363	-3.369209	-1.926083
N	-2.566652	0.015658	-0.025443
H	-2.404460	-0.847700	-0.530985
C	-3.956176	0.289872	0.297358
C	-4.789929	-0.882587	-0.180390
H	-4.097215	0.425092	1.373416
H	-4.305673	1.207168	-0.185777
O	-4.329778	-1.848799	-0.739076
O	-6.084351	-0.704440	0.094821
H	-6.582615	-1.471804	-0.229167
H	4.907084	-3.291980	-0.340116

$[GF_{\beta}{}^{\bullet}G]^{+}$

C	1.933443	-2.487674	-0.202885
H	2.877345	0.196652	1.641329
C	4.222032	-1.162497	0.655111
C	3.188803	-2.966250	-0.560807
H	1.044268	-3.025596	-0.519603
H	5.112162	-0.655410	1.008060
C	4.335696	-2.301017	-0.135088
H	3.273258	-3.864946	-1.159641
N	-2.632948	-0.121700	-0.158866
H	-2.650444	-1.019978	-0.631523
C	-3.920568	0.394791	0.265651
C	-4.970497	-0.624196	-0.122429
H	-3.942834	0.556578	1.345985
H	-4.138829	1.351287	-0.215334
O	-4.714101	-1.652816	-0.684653
O	-6.182807	-0.227239	0.237742
H	-6.829449	-0.898931	-0.025516
H	-0.185245	2.160774	0.714272
H	5.313716	-2.679829	-0.405360

$[GF_{\pi}{}^{\bullet}G]^{+}$

N	1.829578	4.020979	-0.160067
C	2.723063	2.914522	-0.417713
H	1.763095	4.669237	-0.932605
H	2.061910	4.534828	0.678660
C	1.974968	1.580385	-0.426074
H	3.227503	2.984061	-1.384338
H	3.507376	2.815747	0.336544
O	2.595150	0.521986	-0.637817
N	0.672866	1.620914	-0.199522
H	0.263219	2.525891	0.046788
C	-0.166156	0.450479	-0.208287
H	-0.179649	0.020463	-1.215775
C	0.329695	-0.616859	0.799251
C	-1.581191	0.920960	0.174558
H	0.393261	-0.157425	1.787849
H	-0.423660	-1.410306	0.850822
C	1.636777	-1.242916	0.417258
O	-1.756468	2.017116	0.663210
C	2.714628	-1.282631	1.352171
C	1.730390	-2.034976	-0.770432
H	2.627671	-0.714896	2.271603
C	3.842961	-2.011478	1.080855
C	2.862949	-2.758309	-1.035944
H	0.897376	-2.047358	-1.464457
H	4.665921	-2.032979	1.783689
C	3.929139	-2.740581	-0.119487
H	2.940304	-3.346334	-1.941275
N	-2.560601	0.028545	-0.049498
H	-2.400353	-0.849318	-0.529121
C	-3.938402	0.332938	0.271450
C	-4.782418	-0.851962	-0.138895
H	-4.066466	0.519646	1.340653
H	-4.284414	1.225973	-0.256388
O	-4.335822	-1.840896	-0.654090
O	-6.065306	-0.654485	0.140589
H	-6.572026	-1.430177	-0.140986
H	4.827176	-3.307329	-0.335459

$[GF_{\beta}{}^{\bullet}G]^{+}$

N	3.349960	-2.978194	1.495931
C	2.750177	-3.547722	0.296729
H	4.305851	-2.677941	1.349597
H	3.337712	-3.619508	2.279290
C	1.560417	-2.729739	-0.170956
H	3.443564	-3.593931	-0.549132
H	2.389819	-4.569466	0.448054
O	0.968940	-3.120576	-1.234996
N	1.224865	-1.687872	0.556024
H	1.880069	-1.580383	1.340679
C	0.321365	-0.529948	0.260559
H	0.159217	-0.063961	1.231175
C	0.954405	0.436564	-0.694840
C	-1.037618	-0.973741	-0.299365
H	0.059219	-2.601110	-1.364093
H	1.043158	0.097976	-1.722108
C	1.427425	1.726381	-0.369008
O	-1.128852	-1.854460	-1.176720
C	1.387172	2.289025	0.939844
C	1.983431	2.529304	-1.409653
H	0.980771	1.725413	1.772021
C	1.868408	3.563455	1.180739
C	2.460640	3.800786	-1.155554
H	2.024407	2.126286	-2.415585
H	1.827346	3.971224	2.183900
C	2.407116	4.329336	0.139964
H	2.876498	4.390977	-1.963534
N	-2.103040	-0.316865	0.147092
H	-2.028348	0.412528	0.849955
C	-3.444537	-0.528209	-0.377444
C	-4.380368	0.414088	0.358449
H	-3.485853	-0.326998	-1.451495
H	-3.767532	-1.561952	-0.229787
O	-4.011945	1.182981	1.211521
O	-5.636958	0.274092	-0.063633
H	-6.206491	0.890734	0.424404
H	2.780411	5.326866	0.337175

$[b_2 – H]^{\bullet+}$ (oxazolone)

N	-1.580053	3.012370	-0.023428
C	-2.722310	2.121170	0.104628
H	-1.445737	3.592468	0.795504
H	-1.647457	3.616848	-0.832842
C	-2.249890	0.702454	0.038691
H	-3.267738	2.241069	1.048294
H	-3.469801	2.254519	-0.686399
O	-3.019905	-0.332851	0.129793
N	-0.969101	0.359164	-0.118267
H	-0.236501	1.067376	-0.191376
C	-0.820580	-1.001409	-0.136089
C	0.465843	-1.727945	-0.312967
C	-2.140274	-1.557921	0.024477
H	0.459143	-2.187201	-1.311539
C	1.682375	-0.840983	-0.134148
O	-2.606490	-2.643502	0.083040
C	2.203321	-0.610359	1.144386
C	2.287401	-0.235991	-1.240813
H	1.758450	-1.093641	2.008663
C	3.313189	0.215501	1.311926
C	3.397295	0.591442	-1.070675
H	1.910850	-0.429593	-2.240555
H	3.719217	0.376081	2.303636
C	3.909668	0.818660	0.205272

N	2.578419	-3.110256	1.476796
C	2.031095	-3.463724	0.179842
H	3.550450	-2.833053	1.422306
H	2.506050	-3.867331	2.143960
C	0.987249	-2.464630	-0.261510
H	2.775241	-3.496133	-0.621662
H	1.542119	-4.441178	0.184164
O	0.474105	-2.619760	-1.414052
N	0.668796	-1.503137	0.572106
H	1.215208	-1.562188	1.436443
C	-0.042346	-0.234019	0.253495
H	-0.139449	0.277817	1.213154
C	0.725416	0.605372	-0.725361
C	-1.428326	-0.507428	-0.318774
H	-0.413100	-2.041337	-1.508627
H	0.393005	0.605118	-1.757011
C	1.883880	1.334453	-0.368372
O	-1.571820	-1.307372	-1.257711
C	2.405817	1.362479	0.949496
C	2.567109	2.072888	-1.367977
H	1.913803	0.821113	1.750698
C	3.541739	2.093191	1.243866
C	3.701875	2.797894	-1.063278
H	2.181767	2.064006	-2.381480
H	3.922902	2.110660	2.257569
C	4.196468	2.812948	0.242578
H	4.207774	3.358256	-1.839702
N	-2.441388	0.184298	0.171882
H	-2.324174	0.849135	0.930974
C	-3.782327	0.075160	-0.372948
C	-4.671928	1.008928	0.420587
H	-3.801706	0.353731	-1.429393
H	-4.155206	-0.948640	-0.294248
O	-4.271259	1.688946	1.323978
O	-5.922249	0.967311	-0.016194
H	-6.464555	1.574527	0.509152
H	5.084542	3.385075	0.480184

$[b_2 – H]^{\bullet+}$ (oxazolone)

N	-1.369444	3.010027	-0.000122
C	-2.566185	2.192900	0.000378
H	-1.304300	3.600094	0.819382
H	-1.304993	3.600106	-0.819673
C	-2.160354	0.755426	0.000143
H	-3.205723	2.348292	0.875754
H	-3.206512	2.348359	-0.874409
O	-2.986266	-0.236652	0.000455
N	-0.896600	0.345807	-0.000405
H	-0.115086	1.006600	-0.000662
C	-0.833253	-1.018861	-0.000472
C	0.416631	-1.820041	-0.001067
C	-2.199733	-1.479726	0.000093
H	0.394734	-2.481058	-0.875741
C	1.638460	-0.930627	-0.000469
O	-2.721541	-2.538545	0.000293
C	2.186070	-0.493474	1.206046
C	2.186321	-0.492159	-1.206390
H	1.775825	-0.846259	2.147244
C	3.275569	0.372193	1.205538
C	3.275819	0.373514	-1.204708
H	1.776281	-0.843922	-2.148060
H	3.706959	0.695079	2.145064
C	3.819273	0.806857	0.000708

H	3.868751	1.043630	-1.935164
H	0.471072	-2.571551	0.387018
H	4.778756	1.452357	0.335831

$[b_3 – H]^{\bullet +}$ (oxazolone)

N	-2.372411	2.521736	0.000005
C	-1.123179	3.271946	0.000018
C	0.109996	2.368273	0.000002
N	-0.201369	1.014759	0.000042
C	0.545109	-0.128536	0.000049
C	1.958485	-0.180543	0.000014
O	2.530945	-1.401804	0.000032
N	2.869704	0.786716	-0.000037
C	4.215246	0.243730	-0.000056
H	-1.020701	3.922987	0.873298
H	4.782790	0.533609	-0.889083
C	3.954458	-1.252627	-0.000008
H	4.782835	0.533658	0.888928
O	4.675144	-2.180129	-0.000002
O	1.238848	2.833386	-0.000035
H	-1.228654	0.889844	0.000064
H	2.564752	1.774009	-0.000051
H	-2.939008	2.698868	-0.819846
H	-1.020688	3.923040	-0.873223
H	-2.939083	2.698966	0.819782
C	-0.189801	-1.460079	0.000115
H	0.158072	-2.030903	-0.868020
H	0.157996	-2.030775	0.868367
C	-1.700099	-1.378100	0.000044
C	-2.409537	-1.353596	-1.206396
C	-2.409650	-1.353557	1.206417
C	-3.801916	-1.299329	-1.206565
H	-1.874517	-1.398061	-2.149958
C	-3.802030	-1.299292	1.206453
H	-1.874719	-1.397993	2.150030
C	-4.499659	-1.270717	-0.000089
H	-4.340680	-1.296987	-2.146889
H	-4.340882	-1.296920	2.146726
H	-5.583002	-1.242896	-0.000141

$[b_2 – H]^{\bullet +}$ (diketopiperazine)

N	-3.271643	-0.286151	0.000000
C	-3.191935	1.175272	-0.000004
H	-4.187331	-0.722753	0.000002
C	-1.768891	1.714328	-0.000002
H	-3.698326	1.576419	-0.882800
H	-3.698330	1.576425	0.882787
O	-1.530901	2.889364	-0.000002
N	-0.741887	0.758987	-0.000001
H	0.209722	1.126248	-0.000000
C	-0.897512	-0.593117	0.000000
C	0.315491	-1.497388	-0.000000
C	-2.215925	-1.095889	0.000001
H	0.262370	-2.151581	0.880631
H	0.262370	-2.151580	-0.880633
C	1.641756	-0.762167	0.000000
O	-2.512021	-2.390031	0.000003
C	2.257909	-0.415901	1.208326
C	2.257915	-0.415910	-1.208325
H	1.800397	-0.693793	2.152730
C	3.470740	0.270503	1.206971

H	3.707404	0.697431	-2.143790
H	0.394737	-2.482317	0.872645
H	4.673729	1.472317	0.001160

$[b_3 – H]^{\bullet +}$ (oxazolone)

N	-2.495408	2.288270	0.000017
C	-1.308000	3.126156	0.000002
C	-0.022231	2.305649	-0.000002
N	-0.255336	0.939201	0.000013
C	0.553547	-0.149454	0.000015
C	1.964428	-0.128564	0.000004
O	2.582133	-1.321287	0.000011
N	2.833671	0.870905	-0.000012
C	4.196360	0.379242	-0.000017
H	-1.247657	3.778930	0.874468
H	4.749210	0.683628	-0.891464
C	3.980971	-1.123047	-0.000001
H	4.749223	0.683645	0.891414
O	4.735211	-2.017574	0.000001
O	1.073454	2.824536	-0.000016
H	-1.272340	0.752309	0.000022
H	2.503132	1.843904	-0.000018
H	-3.073046	2.421682	-0.819566
H	-1.247669	3.778920	-0.874472
H	-3.073039	2.421701	0.819601
C	-0.109322	-1.514819	0.000036
H	0.255805	-2.066940	-0.872144
H	0.255780	-2.066902	0.872250
C	-1.615803	-1.447030	0.000013
C	-2.317514	-1.385956	-1.204801
C	-2.317551	-1.385946	1.204804
C	-3.703196	-1.264670	-1.204966
H	-1.779710	-1.446804	-2.145849
C	-3.703233	-1.264660	1.204926
H	-1.779776	-1.446786	2.145869
C	-4.396366	-1.200879	-0.000031
H	-4.241367	-1.234370	-2.144600
H	-4.241433	-1.234352	2.144543
H	-5.476525	-1.118736	-0.000048

$[b_2 – H]^{\bullet +}$ (diketopiperazine)

N	-3.246980	-0.251665	-0.000001
C	-3.143756	1.203007	-0.000002
H	-4.168559	-0.676475	-0.000002
C	-1.714121	1.713958	0.000000
H	-3.636416	1.613939	-0.884510
H	-3.636419	1.613940	0.884504
O	-1.459155	2.877893	0.000001
N	-0.700369	0.745430	0.000001
H	0.256951	1.100583	0.000002
C	-0.883958	-0.596667	0.000001
C	0.300465	-1.531288	0.000003
C	-2.208745	-1.074854	-0.000000
H	0.236256	-2.178535	0.884020
H	0.236256	-2.178539	-0.884011
C	1.623910	-0.802086	0.000001
O	-2.518012	-2.358986	-0.000000
C	2.224793	-0.441378	1.206686
C	2.224792	-0.441381	-1.206686
H	1.767211	-0.727611	2.148560
C	3.418626	0.272386	1.205401

C	3.470746	0.270494	-1.206970
H	1.800407	-0.693809	-2.152729
H	3.944769	0.526542	2.146930
C	4.077001	0.614961	0.000001
H	3.944779	0.526526	-2.146928
H	-1.729536	-2.957270	0.000007
H	5.023032	1.142988	0.000001

TS $[GF_{\alpha}{}^{\bullet}G]^{+} \rightarrow [GF_{\pi}{}^{\bullet}G]^{+}$

N	-2.822110	-3.670010	0.068232
C	-2.459805	-2.943498	-1.142252
H	-2.387380	-4.583863	0.114881
H	-3.822511	-3.793673	0.156919
C	-1.496854	-1.830938	-0.823650
H	-1.993144	-3.561191	-1.917664
H	-3.331101	-2.478157	-1.613097
O	-0.979659	-1.083249	-1.704951
N	-1.144448	-1.572797	0.433211
H	-1.525461	-2.141303	1.185779
C	-0.155512	-0.532971	0.521865
H	-0.317876	-0.367686	-0.840730
C	-0.487915	0.598491	1.480239
C	1.299256	-1.058606	0.568948
H	-0.890943	0.205059	2.418641
H	0.417807	1.154856	1.727475
C	-1.495484	1.496149	0.787279
O	1.470825	-2.167750	1.045672
C	-2.867970	1.386816	1.048330
C	-1.050353	2.438864	-0.160096
H	-3.223439	0.673840	1.784365
C	-3.771136	2.221599	0.402142
C	-1.955697	3.271796	-0.808135
H	0.010503	2.542871	-0.364372
H	-4.828153	2.155341	0.631541
C	-3.317572	3.162618	-0.531988
H	-1.600734	4.006327	-1.520773
N	2.255875	-0.233746	0.135509
H	2.032074	0.666956	-0.274798
C	3.675674	-0.538046	0.233469
C	4.439784	0.661757	-0.296872
H	3.971994	-0.733502	1.267715
H	3.934248	-1.425443	-0.351568
O	3.903587	1.663110	-0.704400
O	5.755921	0.457784	-0.249926
H	6.211875	1.241975	-0.596196
H	-4.026866	3.810127	-1.033614

TS $[GF_{\alpha}{}^{\bullet}G]^{+} \rightarrow [GF_{\beta}{}^{\bullet}G]^{+}$

N	-3.382907	2.480790	-0.655750
C	-2.414188	3.421286	-0.098209
H	-3.730293	2.778038	-1.559052
H	-4.180924	2.341104	-0.047862
C	-1.086938	2.757655	0.244862
H	-2.181593	4.231679	-0.794826
H	-2.759854	3.904637	0.820581
O	-0.171208	3.408580	0.763412
N	-1.000379	1.444015	-0.081032
H	-1.879209	1.090859	-0.468715
C	0.027155	0.529766	0.293757
C	-0.336435	-0.890468	0.570723
C	1.428297	0.896079	0.104402

C	3.418625	0.272383	-1.205404
H	1.767208	-0.727617	-2.148558
H	3.885827	0.541655	2.144521
C	4.014592	0.629859	-0.000002
H	3.885824	0.541650	-2.144525
H	-1.743537	-2.935203	0.000001
H	4.946840	1.180970	-0.000003

TS $[GF_{\alpha}{}^{\bullet}G]^{+} \rightarrow [GF_{\pi}{}^{\bullet}G]^{+}$

N	-4.963098	-0.387984	0.914925
C	-4.578778	0.110542	-0.393345
H	-5.255460	-1.356806	0.885267
H	-5.713508	0.152405	1.324582
C	-3.089041	0.309847	-0.456496
H	-4.841954	-0.550335	-1.225136
H	-5.030702	1.080337	-0.617019
O	-2.476452	0.729453	-1.455999
N	-2.330642	0.044469	0.624910
H	-2.778372	-0.234155	1.495884
C	-0.957742	0.128474	0.342431
H	-1.238384	0.686109	-0.822766
C	-0.054624	0.707004	1.408066
C	-0.352059	-1.155347	-0.349807
H	-0.661872	1.272288	2.119062
H	0.447944	-0.099975	1.952456
C	0.974169	1.604667	0.753606
O	-0.932590	-2.191872	-0.181183
C	0.549688	2.714181	0.010480
C	2.339612	1.327771	0.860625
H	-0.509234	2.946922	-0.058870
C	1.478960	3.539588	-0.611797
C	3.266057	2.159191	0.241353
H	2.666243	0.465679	1.432153
H	1.144577	4.401089	-1.176357
C	2.837395	3.261010	-0.498668
H	4.324961	1.953822	0.341722
N	0.786828	-0.961509	-1.033008
H	1.218213	-0.046151	-0.980673
C	1.646871	-2.105682	-1.253065
C	2.256149	-2.566441	0.065969
H	1.073666	-2.927831	-1.684645
H	2.440679	-1.843177	-1.952097
O	2.059721	-2.026027	1.119723
O	3.025174	-3.632445	-0.114612
H	3.391162	-3.912440	0.737399
H	3.563662	3.905678	-0.978295

TS $[GF_{\alpha}{}^{\bullet}G]^{+} \rightarrow [GF_{\beta}{}^{\bullet}G]^{+}$

N	-3.378198	2.377649	-0.592749
C	-2.405872	3.335530	-0.089481
H	-3.792396	2.676447	-1.465871
H	-4.129647	2.209989	0.064508
C	-1.076152	2.679695	0.232839
H	-2.187447	4.118018	-0.820249
H	-2.719570	3.848549	0.823696
O	-0.155654	3.331508	0.733452
N	-0.986452	1.372813	-0.088086
H	-1.863400	1.002100	-0.461782
C	0.050233	0.478343	0.293247
C	-0.309113	-0.933945	0.580877
C	1.444283	0.856560	0.089464

H	0.466907	-1.487788	0.988133
H	-0.227586	0.126107	1.478361
C	-1.585391	-1.568827	0.271882
O	1.848842	2.122856	0.154539
C	-2.365929	-1.276234	-0.866017
C	-1.997118	-2.623319	1.115984
H	-2.038851	-0.534982	-1.586223
C	-3.524170	-1.997247	-1.130910
C	-3.157874	-3.333097	0.848447
H	-1.401013	-2.872917	1.986993
H	-4.102048	-1.774403	-2.019983
C	-3.930220	-3.018843	-0.272384
H	-3.461993	-4.135384	1.509931
N	2.351719	-0.044565	-0.058472
H	2.090388	-1.007735	-0.247890
C	3.782423	0.222553	-0.117269
C	4.484815	-1.115864	-0.275571
H	4.035662	0.871647	-0.960467
H	4.126336	0.722302	0.791648
O	3.894988	-2.164129	-0.365350
O	5.807063	-0.965365	-0.306646
H	6.225967	-1.834579	-0.417151
H	1.089381	2.760927	0.470347
H	-4.834348	-3.577374	-0.482868

TS $[GF_{\pi}{}^{\bullet}G]^{+} \rightarrow [GF_{\beta}{}^{\bullet}G]^{+}$

N	1.726609	4.232136	-1.034987
C	2.211683	3.675720	0.213524
H	2.467907	4.551913	-1.644354
H	1.060802	4.982996	-0.901958
C	1.670314	2.258109	0.445349
H	3.301922	3.602343	0.238314
H	1.913997	4.252458	1.093534
O	1.940451	1.665808	1.515625
N	0.954777	1.724047	-0.537838
H	0.775697	2.388757	-1.293553
C	0.127623	0.534005	-0.386700
H	0.070696	0.046799	-1.363976
C	0.716093	-0.447445	0.656950
C	-1.300797	0.979986	0.050620
H	1.373353	0.328863	1.312763
H	-0.026303	-0.778900	1.384896
C	1.617047	-1.515151	0.248193
O	-1.485952	2.060555	0.577254
C	2.423847	-1.433639	-0.917275
C	1.737276	-2.665203	1.078036
H	2.366297	-0.558894	-1.553992
C	3.281879	-2.462955	-1.247317
C	2.598212	-3.691297	0.742747
H	1.135344	-2.731175	1.977470
H	3.888325	-2.400132	-2.142655
C	3.374411	-3.596637	-0.420738
H	2.673806	-4.567588	1.374655
N	-2.265106	0.065928	-0.186131
H	-2.077985	-0.808133	-0.664929
C	-3.652798	0.288735	0.179959
C	-4.448100	-0.943040	-0.204938
H	-3.756891	0.472508	1.253167
H	-4.066756	1.163221	-0.331173
O	-3.965229	-1.915566	-0.732094
O	-5.738484	-0.807712	0.111986
H	-6.212898	-1.611674	-0.153802
H	4.052316	-4.399943	-0.684353

H	0.478212	-1.544742	1.009931
H	-0.184547	0.097528	1.474578
C	-1.591403	-1.551985	0.273747
O	1.840839	2.080119	0.110316
C	-2.272553	-1.277756	-0.923915
C	-2.128233	-2.500505	1.159338
H	-1.835481	-0.616199	-1.665276
C	-3.471776	-1.915310	-1.209624
C	-3.327651	-3.130487	0.869117
H	-1.601276	-2.730425	2.078845
H	-3.979790	-1.709356	-2.143661
C	-4.006593	-2.834026	-0.311401
H	-3.734972	-3.856312	1.561894
N	2.366409	-0.077324	-0.057270
H	2.112432	-1.047501	-0.221631
C	3.785249	0.223421	-0.131468
C	4.514541	-1.100255	-0.244077
H	4.017506	0.846048	-0.999304
H	4.116896	0.759570	0.760357
O	3.949091	-2.157480	-0.289721
O	5.825332	-0.922363	-0.289911
H	6.264462	-1.782550	-0.369893
H	1.062479	2.717121	0.432598
H	-4.942389	-3.330193	-0.537078

TS $[GF_{\pi}{}^{\bullet}G]^{+} \rightarrow [GF_{\beta}{}^{\bullet}G]^{+}$

N	0.806575	3.661141	-1.231393
C	-0.097297	3.662510	-0.086369
H	1.746378	3.933199	-0.968744
H	0.499333	4.302521	-1.950956
C	-0.364432	2.262877	0.446786
H	0.249022	4.259742	0.761576
H	-1.073995	4.062943	-0.366826
O	-1.044243	2.047873	1.425277
N	0.226130	1.246578	-0.273206
H	0.624867	1.522405	-1.167787
C	-0.102300	-0.103982	0.049513
H	-0.366621	-0.111177	1.111234
C	-1.222878	-0.756967	-0.767936
C	1.047608	-1.097696	-0.092058
H	-1.112618	-0.616445	-1.846616
H	-0.644388	-1.895603	-0.657158
C	-2.581987	-0.801988	-0.322324
O	0.702987	-2.256783	-0.399755
C	-3.594344	-1.129990	-1.276100
C	-2.972069	-0.574501	1.031319
H	-3.302935	-1.302624	-2.306082
C	-4.908915	-1.235106	-0.898514
C	-4.293791	-0.673275	1.395825
H	-2.238428	-0.279218	1.769577
H	-5.669875	-1.488241	-1.625591
C	-5.266061	-1.009527	0.441550
H	-4.588240	-0.484489	2.420219
N	2.286310	-0.727667	0.131154
H	2.488995	0.243738	0.357880
C	3.414900	-1.637265	0.049849
C	4.676907	-0.813344	0.199901
H	3.374403	-2.391304	0.839779
H	3.423491	-2.159762	-0.909467
O	4.671469	0.374431	0.366522
O	5.757619	-1.576746	0.126800
H	6.547143	-1.025359	0.233261
H	-6.304340	-1.091378	0.740402

TS $[GF_{\pi}{}^{\bullet}G]^{+} \rightarrow [G_{\alpha}{}^{\bullet}FG]^{+}$

N	1.311415	4.350643	-0.466088
C	0.774335	3.168117	-0.886595
H	1.251171	4.579546	0.523927
H	1.804841	4.977077	-1.087950
C	-0.149356	2.510739	0.131725
H	1.535483	2.152034	-0.691593
H	0.549053	3.115772	-1.952187
O	-0.165345	2.904244	1.275363
N	-0.658045	1.306402	-0.300604
H	-0.632506	1.163943	-1.303526
C	-0.239797	0.132743	0.457695
H	-0.530740	0.279666	1.500031
C	-0.899897	-1.153491	-0.096432
C	1.313300	0.054224	0.404279
H	-0.531812	-1.335496	-1.110960
H	-0.562786	-2.004493	0.503106
C	-2.412558	-1.090669	-0.089573
O	2.015846	0.868367	-0.252475
C	-3.122384	-1.037650	-1.291631
C	-3.122661	-1.094915	1.115852
H	-2.588464	-1.059595	-2.236847
C	-4.515774	-0.980975	-1.291626
C	-4.513315	-1.036508	1.118585
H	-2.593173	-1.154106	2.062468
H	-5.053584	-0.947748	-2.231931
C	-5.213120	-0.976778	-0.086569
H	-5.051291	-1.045420	2.059289
N	1.904809	-0.917342	1.094536
H	1.315518	-1.613456	1.529118
C	3.329388	-1.198014	1.006398
C	3.661618	-1.989192	-0.259962
H	3.891125	-0.263472	1.000811
H	3.638217	-1.766950	1.884265
O	2.856188	-2.321562	-1.087032
O	4.973444	-2.251863	-0.303698
H	5.167854	-2.758399	-1.108696
H	-6.295904	-0.936099	-0.084374

TS $[G_{\alpha}{}^{\bullet}FG]^{+} \rightarrow [GF_{\beta}{}^{\bullet}G]^{+}$

N	3.635556	-2.812219	-0.742095
C	2.888747	-2.133393	0.202460
H	4.563074	-2.480106	-0.954190
H	3.137892	-3.245153	-1.507448
C	1.526936	-2.589956	0.457067
H	3.458790	-1.777927	1.059667
H	2.297850	-0.984120	-0.180274
O	0.904141	-3.257360	-0.390402
N	0.802851	-1.854249	1.411593
H	1.390357	-1.539591	2.176278
C	0.172663	-0.658818	0.749874
H	-0.035291	0.054768	1.545541
C	1.074907	-0.018153	-0.311897
C	-1.167438	-1.081048	0.159429
H	-0.448563	-2.808926	-0.391085
H	0.864905	-0.322702	-1.336245
C	1.542906	1.349774	-0.178773
O	-1.334372	-2.219939	-0.399866
C	1.704353	1.993858	1.069615

TS $[GF_{\pi}{}^{\bullet}G]^{+} \rightarrow [G_{\alpha}{}^{\bullet}FG]^{+}$

N	1.230991	4.281096	-0.667575
C	0.671194	3.083052	-0.997641
H	1.338780	4.492110	0.326226
H	1.557239	4.946587	-1.358618
C	-0.056310	2.435277	0.194781
H	1.466749	2.162302	-0.898191
H	0.262728	3.022190	-2.005629
O	0.169872	2.852098	1.299521
N	-0.663395	1.256571	-0.107042
H	-0.802935	1.060533	-1.091755
C	-0.196783	0.108993	0.660666
H	-0.463666	0.251584	1.710067
C	-0.815376	-1.189685	0.106651
C	1.337890	0.050259	0.518884
H	-0.365945	-1.407500	-0.867509
H	-0.537582	-2.016636	0.765392
C	-2.316748	-1.090406	-0.009593
O	1.959277	0.763629	-0.285998
C	-2.925296	-1.077545	-1.262660
C	-3.110488	-0.988788	1.133580
H	-2.319535	-1.184745	-2.157592
C	-4.308291	-0.958664	-1.374996
C	-4.489664	-0.869512	1.023910
H	-2.651662	-1.012370	2.117695
H	-4.771913	-0.959745	-2.354020
C	-5.090984	-0.851243	-0.232296
H	-5.097630	-0.797808	1.917517
N	1.979590	-0.837362	1.280759
H	1.428623	-1.469704	1.843791
C	3.349219	-1.197673	0.983467
C	3.414625	-1.966416	-0.331740
H	3.965366	-0.301933	0.899595
H	3.753121	-1.812887	1.786965
O	2.459572	-2.247307	-0.996601
O	4.674396	-2.276587	-0.626808
H	4.692178	-2.771579	-1.458954
H	-6.167085	-0.763107	-0.316964

TS $[G_{\alpha}{}^{\bullet}FG]^{+} \rightarrow [GF_{\beta}{}^{\bullet}G]^{+}$

N	3.503665	-2.871158	-0.823971
C	2.820847	-2.159350	0.143133
H	4.411864	-2.534646	-1.102295
H	2.946898	-3.271167	-1.566684
C	1.459014	-2.573093	0.438239
H	3.423728	-1.808891	0.978857
H	2.238083	-1.019753	-0.234867
O	0.801296	-3.219440	-0.388906
N	0.779899	-1.832253	1.415707
H	1.403308	-1.520077	2.152879
C	0.155137	-0.642494	0.762244
H	-0.022553	0.089750	1.549097
C	1.027252	-0.035789	-0.336248
C	-1.201048	-1.046862	0.208618
H	-0.512371	-2.789669	-0.330823
H	0.767702	-0.326869	-1.354136
C	1.566926	1.303218	-0.193124
O	-1.403938	-2.189167	-0.310360
C	1.845988	1.873486	1.064506

C	1.909131	2.064290	-1.343656
H	1.458701	1.480335	1.993340
C	2.192947	3.290313	1.143569
C	2.396809	3.359712	-1.263842
H	1.797690	1.589992	-2.313010
H	2.308293	3.766977	2.109803
C	2.540302	3.980707	-0.020602
H	2.663070	3.891594	-2.169519
N	-2.180764	-0.249051	0.201470
H	-2.090779	0.664063	0.643737
C	-3.500415	-0.523489	-0.360517
C	-4.372893	0.687424	-0.068934
H	-3.441501	-0.691965	-1.438756
H	-3.936782	-1.418412	0.089786
O	-3.970174	1.659464	0.519627
O	-5.603025	0.518471	-0.545039
H	-6.137625	1.304072	-0.343426
H	2.920127	4.993315	0.041710

TS $[GF_\alpha{}^\bullet G]^+ \rightarrow [b_2 - H]^{\bullet +}$ (oxazolone)

N	2.211485	2.986628	-1.166342
C	0.998238	3.544764	-0.577955
H	3.046219	3.255937	-0.659699
H	2.333632	3.259040	-2.133390
C	0.148530	2.469887	0.078333
H	1.187434	4.303132	0.188547
H	0.368932	4.030156	-1.330262
O	-0.874400	2.674089	0.721770
N	0.579658	1.170284	-0.073747
H	1.464560	1.018233	-0.561849
C	-0.079794	0.173274	0.572806
C	0.392259	-1.264750	0.564394
C	-1.315412	0.483173	1.176222
H	-0.195772	-1.806502	-0.185950
C	1.875744	-1.397168	0.288999
O	-2.038012	0.321454	2.062042
C	2.808042	-1.203438	1.315450
C	2.329664	-1.716590	-0.994894
H	2.468313	-0.972401	2.320236
C	4.171216	-1.328537	1.060491
C	3.695485	-1.840685	-1.249422
H	1.615464	-1.892743	-1.793117
H	4.884299	-1.191134	1.864893
C	4.616836	-1.645377	-0.223003
H	4.036095	-2.102491	-2.244288
N	-2.721039	0.829724	-0.537924
H	-2.302946	0.622812	-1.441122
C	-3.923730	0.029080	-0.312355
C	-3.626541	-1.433977	-0.577357
H	-4.247478	0.140976	0.726767
H	-4.767317	0.334118	-0.942394
O	-2.549508	-1.849909	-0.934224
O	-4.705299	-2.192005	-0.371905
H	-4.484585	-3.118704	-0.558656
H	-2.911286	1.825005	-0.468096
H	0.130762	-1.707523	1.530832
H	5.677463	-1.751314	-0.418090

TS $[GF_\alpha{}^\bullet G]^+ \rightarrow [b_3 - H]^{\bullet +}$ (oxazolone)

N	-2.730147	2.665726	0.151510
C	-1.473080	3.374356	-0.059472

C	1.882040	2.045105	-1.348081
H	1.647297	1.322466	1.978451
C	2.398214	3.140335	1.158898
C	2.432248	3.311872	-1.248066
H	1.679864	1.617928	-2.324438
H	2.607064	3.565362	2.132895
C	2.690585	3.865752	0.004652
H	2.658965	3.873018	-2.146160
N	-2.183219	-0.185191	0.236859
H	-2.062201	0.739586	0.646500
C	-3.502093	-0.461214	-0.313026
C	-4.349598	0.773869	-0.078366
H	-3.442756	-0.676947	-1.382172
H	-3.954359	-1.324781	0.179686
O	-3.931812	1.756877	0.466787
O	-5.574335	0.608066	-0.549397
H	-6.095686	1.408382	-0.384433
H	3.119962	4.856814	0.082206

TS $[GF_\alpha{}^\bullet G]^+ \rightarrow [b_2 - H]^{\bullet +}$ (oxazolone)

N	2.263218	2.893429	-0.928730
C	1.062514	3.453054	-0.326940
H	3.073219	2.995693	-0.329014
H	2.482471	3.326080	-1.816384
C	0.171895	2.364893	0.241822
H	1.244223	4.162365	0.485078
H	0.462192	3.982133	-1.071854
O	-0.793374	2.542721	0.946969
N	0.533238	1.067366	-0.106240
H	1.407241	0.950546	-0.626194
C	-0.119807	0.001853	0.399151
C	0.353506	-1.409659	0.141178
C	-1.306881	0.189390	1.104518
H	-0.107863	-1.750860	-0.790830
C	1.862245	-1.449300	0.062141
O	-2.101446	0.026184	1.901253
C	2.626990	-1.384944	1.228319
C	2.499389	-1.501491	-1.176542
H	2.135390	-1.352981	2.195891
C	4.014038	-1.379000	1.154526
C	3.890467	-1.493731	-1.249707
H	1.909855	-1.569403	-2.085533
H	4.602279	-1.343460	2.063366
C	4.646952	-1.430840	-0.085598
H	4.379373	-1.548614	-2.214648
N	-2.717586	0.975733	-0.684809
H	-2.368525	0.853362	-1.630925
C	-3.945911	0.220040	-0.480513
C	-3.648302	-1.264499	-0.469557
H	-4.372873	0.473529	0.493902
H	-4.724354	0.407265	-1.227224
O	-2.540713	-1.737947	-0.500038
O	-4.764237	-1.979526	-0.414759
H	-4.540685	-2.921573	-0.392749
H	-2.873371	1.966571	-0.527302
H	-0.033701	-2.058532	0.929981
H	5.728628	-1.433870	-0.141300

TS $[GF_\alpha{}^\bullet G]^+ \rightarrow [b_3 - H]^{\bullet +}$ (oxazolone)

N	-2.927394	2.172778	-0.235764
C	-1.774784	3.033670	-0.025794

C	-0.255792	2.446041	-0.067997
N	-0.570693	1.126118	0.160726
C	0.143142	-0.048275	0.279759
C	1.538794	-0.252422	-0.045042
O	2.079979	-1.378210	0.141370
N	2.323135	0.743878	-0.548567
C	3.672857	0.400127	-0.900087
H	-1.283932	4.111774	0.725550
H	3.746085	-0.189102	-1.816073
C	4.298777	-0.396574	0.223397
H	4.273750	1.307503	-1.002807
O	4.764803	-0.293701	1.262797
O	0.867947	2.901180	-0.256535
H	-1.589108	1.040933	0.302965
H	1.989095	1.724884	-0.510628
H	-3.330945	2.675829	-0.663248
O	4.483371	-2.079551	-0.487513
H	4.988294	-2.656972	0.107981
H	3.523131	-2.299471	-0.406668
H	-1.443057	3.925089	-1.004520
H	-3.256841	3.024931	0.937253
C	-0.624558	-1.228764	0.828375
H	-0.069096	-2.133125	0.579241
H	-0.610534	-1.161370	1.924972
C	-2.062889	-1.334130	0.353391
C	-2.346146	-1.610627	-0.990687
C	-3.124927	-1.185254	1.249168
C	-3.662271	-1.731510	-1.426900
H	-1.532982	-1.745669	-1.697415
C	-4.445027	-1.309588	0.814384
H	-2.921990	-0.985137	2.296502
C	-4.715827	-1.580801	-0.523916
H	-3.867162	-1.955626	-2.467473
H	-5.257706	-1.203015	1.523582
H	-5.740180	-1.684726	-0.862085

TS $[GF_{\alpha}{}^{\bullet}G]^{+} \rightarrow [b_2 - H]^{\bullet+}$ (diketopiperazine)

N	1.958725	1.269047	-0.159218
C	1.605580	2.505640	-0.873997
H	2.351801	1.528621	0.743421
H	0.786425	-0.935574	1.703379
C	0.470942	3.278593	-0.215061
H	2.477155	3.158152	-0.922503
H	1.318359	2.264014	-1.899059
O	0.256808	4.456865	-0.396161
N	-0.332337	2.524770	0.640888
H	-1.055335	3.068630	1.099746
C	-0.241397	1.166893	0.929467
C	-1.403786	0.508445	1.628040
C	0.788892	0.432882	0.112874
H	-1.056735	-0.132404	2.447745
H	-1.995814	1.289197	2.117957
C	-2.302608	-0.321422	0.712348
O	0.306051	0.044172	-1.166118
C	-2.513974	-1.680777	0.962492
C	-2.930628	0.263931	-0.397400
H	-2.063833	-2.144006	1.835794
C	-3.328640	-2.442962	0.124309
C	-3.741804	-0.498039	-1.237454
H	-2.807456	1.324125	-0.596516
H	-3.493905	-3.492015	0.341705
C	-3.939734	-1.854683	-0.980539
H	-4.229105	-0.028349	-2.083948

C	-0.467017	2.248550	0.031197
N	-0.621430	0.921661	-0.294171
C	0.206186	-0.168670	-0.255758
C	1.644324	-0.157175	-0.297434
O	2.287185	-1.236841	-0.280210
N	2.376974	0.990437	-0.366754
C	3.792707	0.799319	-0.463167
H	-1.812023	3.625718	0.892493
H	4.119483	0.502536	-1.460243
C	4.183056	-0.302436	0.499278
H	4.325819	1.696577	-0.144232
O	4.373231	-0.522025	1.598929
O	0.578729	2.786422	0.347437
H	-1.619866	0.718030	-0.446208
H	1.950122	1.881809	-0.078820
H	-3.565334	2.534536	-0.931369
O	4.827732	-1.616174	-0.587894
H	5.346280	-2.271320	-0.096801
H	3.982557	-2.023804	-0.859539
H	-1.663534	3.743604	-0.848957
H	-3.449602	1.996974	0.613978
C	-0.448052	-1.531835	-0.275794
H	-0.213848	-2.018562	-1.229137
H	0.039718	-2.140733	0.491228
C	-1.940012	-1.482066	-0.056690
C	-2.819900	-1.514663	-1.137436
C	-2.453369	-1.328850	1.233479
C	-4.192525	-1.394793	-0.935030
H	-2.431532	-1.639770	-2.143218
C	-3.822849	-1.209681	1.437791
H	-1.775526	-1.311053	2.081518
C	-4.694919	-1.238827	0.351493
H	-4.867066	-1.431627	-1.781863
H	-4.211552	-1.107479	2.444068
H	-5.763240	-1.155882	0.511024

TS $[GF_{\alpha}{}^{\bullet}G]^{+} \rightarrow [b_2 - H]^{\bullet+}$ (diketopiperazine)

N	2.361292	-1.102985	-0.147701
C	3.083492	-0.096222	-0.924233
H	2.883499	-1.326651	0.696308
H	0.733992	-1.729001	1.993986
C	3.112091	1.275406	-0.272764
H	4.111914	-0.418234	-1.078272
H	2.616081	-0.002764	-1.907182
O	3.936629	2.119072	-0.504921
N	2.078144	1.510153	0.632356
H	2.124887	2.417780	1.084144
C	1.058322	0.640537	0.986362
C	-0.150144	1.203171	1.674999
C	1.035153	-0.647587	0.215089
H	-0.538775	0.484676	2.404649
H	0.153832	2.079199	2.257741
C	-1.277319	1.587564	0.721110
O	0.288347	-0.604092	-0.976834
C	-2.589936	1.195381	0.983823
C	-1.013611	2.330700	-0.434903
H	-2.814784	0.645988	1.893678
C	-3.620115	1.527308	0.107165
C	-2.042194	2.658970	-1.313315
H	-0.003506	2.670593	-0.645056
H	-4.636176	1.225189	0.331119
C	-3.346982	2.253799	-1.046274
H	-1.824768	3.241562	-2.200385

N	1.311826	-0.759542	0.857557
C	1.533700	-1.976069	0.091648
C	3.011026	-2.146790	-0.104486
H	1.145279	-2.871836	0.584312
H	1.074031	-1.925016	-0.903576
O	3.798589	-1.263828	0.345235
O	3.458240	-3.175967	-0.725641
H	4.430050	-3.177745	-0.813890
H	-0.658404	-0.051125	-1.130737
H	-4.575997	-2.444116	-1.629787
H	3.183067	-0.574267	0.759404

N	0.446362	-1.772709	1.021089
C	-0.981495	-2.026471	0.847793
C	-0.991392	-3.108897	-0.210313
H	-1.423733	-2.431997	1.761163
H	-1.572417	-1.171858	0.514569
O	0.115983	-3.596524	-0.489953
O	-2.099188	-3.480415	-0.736896
H	-1.974851	-4.181264	-1.400822
H	-0.219515	0.217935	-1.027816
H	-4.147040	2.514252	-1.727941
H	0.741375	-2.942902	0.171516
