# Supplementary figures and images for: Structure and fragmentation chemistry of the peptide radical cations of glycylphenylalanylglycine (GFG)
Source: PLoS One. 2024 Aug 13;19(8):e0308164. doi: 10.1371/journal.pone.0308164 (PMC11321575; doi:10.1371/journal.pone.0308164)

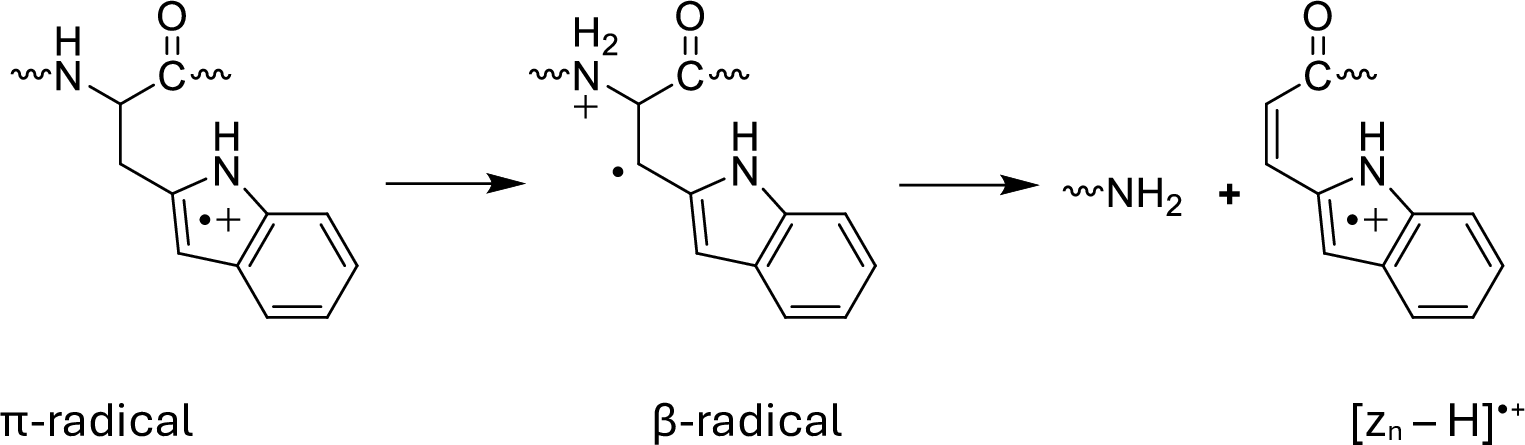

Supplement: S1 Scheme — (TIF) [file pone.0308164.s004.tif]
